# Crustacean Processing By-Products as Sustainable Sources of Bioactive Compounds: A Comprehensive Review of Conventional and Emerging Extraction Technologies and Applications

**DOI:** 10.3390/ijms27156959

**Published:** 2026-08-03

**Authors:** Akanksha R. Gautam, Soottawat Benjakul, Rattikarn Boonchoosri, Vijay Kumar Reddy Surasani, Nilesh Nirmal, Seow Lay Jing, Avtar Singh

**Affiliations:** 1 International Center of Excellence in Seafood Science and Innovation (ICE-SSI), Faculty of Agro-Industry, Prince of Songkla University, Hat Yai 90110, Songkhla, Thailand; 2 Department of Food and Nutrition, Kyung Hee University, Seoul 02447, Republic of Korea; 3 Department of Fish Processing Technology, College of Fisheries, Guru Angad Dev Veterinary and Animal Sciences University, Ludhiana 141004, PB, India; 4 Institute of Nutrition, Mahidol University, 999 Phutthamonthon 4 Road, Salaya 73170, Nakhon Pathom, Thailand; 5 Department of Pharmaceutical Chemistry, Faculty of Pharmacy and Health Sciences, Universiti Kuala Lumpur, Royal College of Medicine Perak, Ipoh 30450, Perak, Malaysia

**Keywords:** seafood, bioactive compounds, novel processing methods, waste utilization

## Abstract

Crustacean processing industries, particularly those that process shrimp, crab, and lobster, generate substantial quantities of biological waste, consisting primarily of shells, heads, and exoskeletons. These by-products contribute significantly to environmental pollution due to their high organic load, slow degradability, and improper disposal practices. Nevertheless, they represent valuable reservoirs of bioactive compounds such as chitin, proteins, polyunsaturated fatty acids, carotenoids, and minerals, as well as biologically active enzymes and enzyme inhibitors, which possess immense potential in food, pharmaceutical/cosmetic, biomedical and environmental sectors. Unlike previous studies that primarily focus on individual components or specific extraction techniques, this review comparatively evaluates both conventional extraction techniques (acid–alkali treatment and solvent extraction) and emerging green approaches (supercritical fluid extraction, enzymatic hydrolysis, pulsed electric fields, ultrasound-assisted extraction, cold plasma, microwave-assisted extraction and high-pressure processing), highlighting their efficiencies, sustainability, and influence on compound quality. The review study further explores the diverse applications of the recovered constituents in food preservation, nutraceutical development, biodegradable packaging, and functional ingredient formulation. Moreover, current challenges concerning process optimization, industrial scalability, economic feasibility, and environmental impact are critically evaluated.

## 1. Introduction

Waste, in a broader sense, refers to any material that has been discarded, disused, or has lost its value in its original form. Among industrial sectors, food processing, agriculture, and marine industries contribute substantially to global waste generation [1]. In the seafood industry, particularly shrimps, prawns, crabs and lobsters are being processed on large scale due to their popularity worldwide. Thus, to meet the demand, a substantial amount of waste is accumulated in the form of shells, heads, and exoskeletons [2]. Although other crustaceans such as krill and barnacles are rarely utilized, their contribution to waste generation is comparatively minor, probably because of their limited commercial processing. It has been estimated that around 6–8 million tons of crustacean waste is generated globally [3]. Cephalothorax, shells, and exoskeletons constitute 50–70% of total animal weight [4]. Depending upon conditions such as processing expertise and species, 45–60% of shrimp is discarded as waste [5]. Annually, the lobster processing industry generates approximately 50,000 tons of by-product, which is valued at around $7.5 million [6]. Additionally, the crab industry produces around 8 million tons of waste annually, which is approximately 70% of total crab weight [7]. Uncontrolled disposal of this waste can lead to environmental pollution because it consists of high organic loads and slow degradation. With a right scientific approach, this waste can be converted into high-value-added products such as chitin (15–40%), protein (20–40%), minerals (20–50%; mainly calcium carbonate), along with some minor components like lipids and pigments (astaxanthin). However, the amount or availability of those products depends on species, season, age, and other external factors [8]. In addition to these components, crustacean by-products also serve as important sources of bioactive enzymes (e.g., proteases and lipases) and enzyme inhibitors, which exhibit significant functional and regulatory roles. These biomolecules are involved in catalytic processes such as protein and lipid hydrolysis, while enzyme inhibitors can modulate enzymatic activity, offering potential applications in food preservation, pharmaceuticals, and therapeutic formulations. Protein sourced from crustaceans, especially from shells, is known to be rich in essential amino acids, offering appreciably strong nutritional profile, is applicable in both food and feed industries, and has an annual estimated market value of $100 million [9]. Furthermore, lipids extracted from crustacean waste are gaining attention due to their valuable components such as omega-3 fatty acids (EPA and DHA), phospholipids, and carotenoids, which are beneficial in human nutrition and health. These lipids can be utilized in nutraceuticals, functional foods, and cosmetic formulations. Additionally, astaxanthin, a red color pigment known for its cardiovascular, wound healing, anticancer benefits and antioxidant properties, can be used as a food colorant [10]. Commercial astaxanthin production is dominated by synthetic methods, despite the availability of natural, bioavailable sources [11].

**Figure 1:**
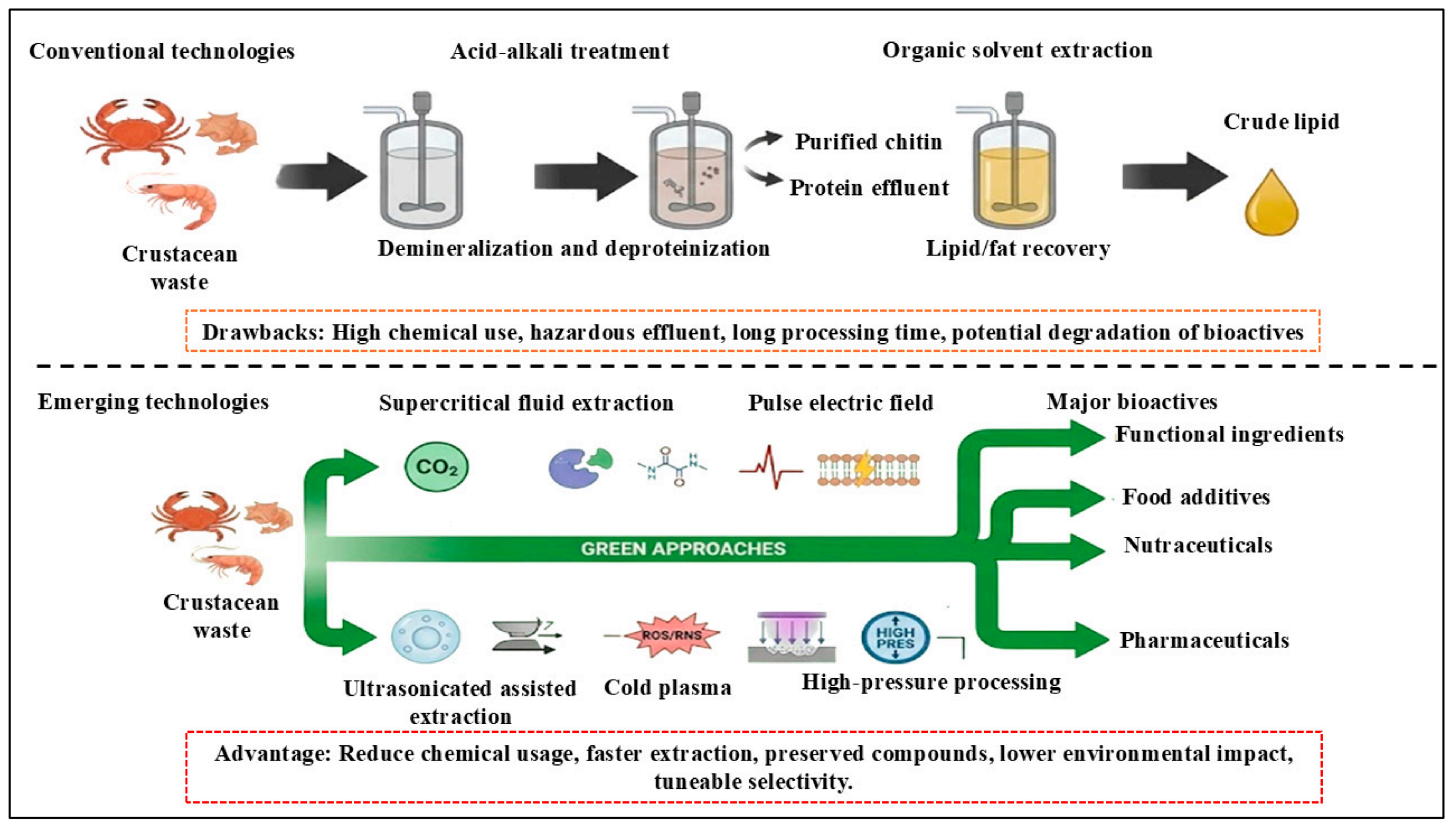
Comparative overview of conventional and green extraction technologies used for the extraction of bioactive compounds from crustacean processing by-products.

Over the past decade, growing concerns about the global food security, regenerative economy, and advances in extraction technologies have driven increasing interest in green and sustainable waste-management strategies. Advanced and innovative valorization techniques such as enzymatic extraction, biorefinery approaches and microbial fermentation of crustacean waste maximize the product recovery and minimize the environmental effect [12]. Various novel pretreatments, such as ultrasound, pulsed electric fields, and cold plasma, have been implemented to enhance the extraction of bioactive compounds (Figure 1). The review provides a comprehensive analysis of crustacean waste utilization, focusing on its composition, innovative extraction methods, and applications in the food industry. Moreover, it examines challenges associated with commercial-scale implementation. The review highlights future sustainable strategies for realizing the full potential of crustacean by-products.

## 2. Valuable Compounds Derived from Crustacean By-Products

Crustacean processing by-products constitute a complex biological matrix composed of structurally integrated biopolymers, proteins, lipids, minerals, and associated bioactive compounds. These residues, primarily including exoskeletons, cephalothorax, hepatopancreas, and processing effluents, serve as important sources of high-value biomolecules with diverse physicochemical and biological functionalities. The major constituents encompass chitin and its derivatives (chitosan and chitooligosaccharide), protein fractions and derived bioactive peptides, lipid classes enriched in long-chain polyunsaturated fatty acids, carotenoids such as astaxanthin, and inorganic components predominantly calcium carbonate. In addition, crustacean by-products contain a wide array of endogenous enzymes and enzyme inhibitors with significant catalytic and regulatory potential (Table 1).

### 2.1. Chitin and Its Derivatives

Chitin is a linear aminopolysaccharide composed of N-acetyl-D-glucosamine (β-(1→4)-N-acetyl-d-glucosamine) and is the second most abundant polysaccharide after cellulose [13]. Chitin serves as a primary structural component in the exoskeletons of crustaceans, where it forms a complex matrix closely associated with calcium carbonate and proteins, contributing to the rigidity and strength of the shell [14]. In shrimp, lobster and crab, chitin is predominantly present in the α-chitin form, which is characterized by an anti-parallel chain arrangement that confers high crystallinity, rigidity, and thermal stability. The highly crystalline nature of α-chitin provides excellent mechanical strength and stability. Reported chitin contents include approximately 69.8% in *Nephrops* (lobster), 24% in *Homarus* (lobster), 60–75% in *Chionoecetes opilio* (crab), 17.8% in *Euphausia superba* (krill), 58.3% in *Crangon crangon* (shrimp), and 26% in *Lepas* (goose barnacle) [15]. The substantial variation in chitin content among crustacean species reflects differences in shell composition, demineralization degree, and biological characteristics. Crustacean shells contain tightly packed chitin fibers intricately interwoven with proteins through covalent bonding. This chitin-protein matrix is further strengthened by the deposition of mineral salts, primarily calcium carbonate, enhancing the shell’s rigidity and structural integrity. In general, efficient recovery of chitin requires demineralization and deproteinization to remove calcium carbonate and proteins from the chitin–protein–mineral matrix [16,17,18]. Acid treatment removes shell minerals, mainly calcium carbonate, by dissolving them into soluble salts and releasing carbon dioxide. Then alkali treatment breaks and removes the proteins bound to the chitin matrix by hydrolyzing peptide bonds. This sequential acid–alkali process leaves purified chitin with minimal damage to its structure. Extraction time and the concentration of acid or alkali affect the demineralization and deproteinization of shells. The quality of extracted chitin is generally evaluated based on parameters such as ash content, residual protein content, degree of acetylation, molecular weight, and crystallinity index, which determine its suitability for further conversion into chitosan and other value-added derivatives. For example, Mittal et al. [18] extracted chitin and chitosan from shrimp shell various HCl concentrations (0.5–1.0 M) at room temperature for demineralization (for various times 1–4 h) and NaOH treatment conditions (1 M, 60–70 °C for 60–80 min) for deproteinization. Among the tested conditions, demineralization with 1 M HCl at a shell-to-solution ratio of 1:20 (*w*/*v*) for 2 h, followed by deproteinization with 1 M NaOH at 70 °C using a shell-to-solution ratio of 1:30 (*w*/*v*) for 80 min, resulted in chitin with the lowest ash content (1.04%) and residual protein content (67.06 mg/g). In addition, the same treatment yielded 29.96% of the chitin from shrimp shells. Conventional acid–alkali extraction is widely used due to its simplicity and efficiency, but it can degrade polymers and generate harmful effluents. Thus, alternative, enzymatic and biological methods have been employed, which use fewer harsh chemicals but require more time and higher costs. For example, Dhanabalan et al. [16] explored enzymatic hydrolysis for protein removal from *Acetes* shrimp shells. They noticed that alcalase achieved 93.68% protein removal under optimized conditions (pH 8.0, 53.3 °C) for 160 min, producing chitin with a degree of acetylation ranging from 73.87 to 97.73%. However, enzymatic deproteinization alone left residual proteins and minerals, requiring additional dilute alkali and acid treatments to obtain sufficiently purified chitin [16]. Although this hybrid enzymatic–chemical process reduces dependence on harsh extraction conditions, the additional purification and washing steps increase processing costs, water consumption, and effluent generation, thereby limiting its economic attractiveness for large-scale, low-value applications. Table 1 provides an overview of the chemicals and enzymes used for the extraction of chitin and its derivatives such as chitosan and chitooligosaccharide from the crustacean waste. Despite numerous studies on chitin purification and extraction, universally accepted standards have yet to be established, and direct applications remain limited by poor solubility in most the solvents.

**Table 1:** Chemical and non-thermally assisted recovery techniques for bioactives from crustacean by-products.

Source	Bioactives	Recovery Technique	Extraction Performance	Advantages/Limitations (Comparative Remarks)	Reference
*Litopenaeus vannamei* shells	Protein isolate and hydrolysates	Demineralization using 1 M HCl (1:20, room temperature) followed by alkaline solubilization of protein using 1 M NaOH (1:30, 60 °C). Ultrasound-assisted enzymatic hydrolysis (2% Alcalase and 3% papain) Amplitude: 60, Time: 15 min, Pulse mode (5 s on/5 s off).	Ultrasonication enhanced DH from 2.58 to 23.16% to 9.28–31.12% (Alcalase) and from 2.17 to 20.48% to 22.71–42.94% (Papain). Maximum DH of 42.94% achieved with 3% Papain.	Enhanced hydrolysis under mild conditions. Enzyme cost and process optimization remain challenges.	[17]
Acetes shrimp shell	Chitin	Alcalase-aided enzymatic deproteinization pH: 8.5, 53.3 °C, enzyme/substrate ratio 1.53, followed by deproteinization (0.5 M NaOH) and demineralization (0.5 M HCl).	93.68% protein removal at 30% DH. DA: 73.87–97.73%.	Efficient protein removal. Multi-step and cost-intensive.	[16]
*Litopenaeus vannamei* Shells	Chitin and chitosan	Demineralization (1 M HCl, 1:20 *w*/*v*, 2 h) and deproteinization (1 M NaOH, 1:30 *w*/*v*, 70 °C, 80 min). Deacetylation at 130 °C for 4 h.	Optimum chitin yield of 29.96%. Chitosan yield of 73.11% with 85.28% DDA.	High yield. Established method. Use of harsh chemicals. Environmental impact.	[18]
Lobster shells	Chitin	Deep eutectic solvents (DES)-assisted extraction using choline chloride combined with organic acids (malonic, malic, and other acids) at 50–100 °C.	Highest chitin purity of 93% obtained using choline chloride–malonic acid DES. Molecular weight varied from 91 to 312 kDa depending on acid type and extraction temperature. CaCO_3_ was simultaneously converted into calcium levulinate.	Green solvent method High purity Scale-up limitations	[19]
Brown Crab shell	Chitin	Isolation via the DES (ChCl/lactic acid) up to 130 °C.	Chitin purity of up to 98% Comparable to commercial and conventionally extracted chitin, while reducing processing time by more than eightfold. ChCl/lactic acid DES exhibited low phytotoxicity (EC_50_ ≥ 1.6 mg/mL).	High purity. Green process. Industrial scale validation lacking.	[20]
Snow crab shell (*Chionoecetes opilio*)	Chitin	Extraction via DES (TEBAC and lactic acid).	Deproteinization rate: 95.51%. Decalcification rate: 96.41%. Chitin purity: 91.15%. Chitin yield: 21.31%.	High efficiency. Solvent optimization required.	[21]
Shrimp shell	Chitin	Choline chloride–malic acid-based natural deep eutectic solvents, assisted by microwave irradiation.	Effective demineralization and deproteinization. Chitin relative crystallinity: 71%.	Fast and efficient. Equipment cost and scale-up issues.	[22]
North Atlantic shrimps (*Pandalus borealis*)	Chitin	Ultrasound-assisted demineralization (0.25 M HCl, 1:40, 40 °C, 4 h) and deproteinization (0.25 M NaOH, 1:40) using ultrasonication at 41 W/cm^2^ for 0–4 h.	Protein content reduced from 39.8% to 7.3% after 4 h ultrasonication. Chitin yield decreased from 16.5% to 11.4% Crystallinity index decreased from 87.6% to 78.5%, while degree of acetylation remained unchanged.	Process intensification. Chitin loss and structural changes.	[23]
Chinese mitten crab shells (*Eriocheir sinensis*)	Chitin	Plasma-assisted extraction combining atmospheric pressure plasma jet pretreatment with HCl demineralization and NaOH deproteinization.	Produced high-purity chitin without chemical decolorization. Crystallinity of 64.58% Thermal stability of 402.9 °C. Molecular weight of 6.89 × 10^5^ g/mol, comparable to commercial chitin.	Solvent-reduced. Advanced green technology; limited scalability and process standardization.	[24]
Crustacean shell waste	Chitin	Atmospheric-pressure dielectric barrier discharge (DBD) cold plasma.	Efficient and rapid protein removal without significant alteration of chitin structure. Solvent-free process generating no liquid or solid waste and reducing chemical requirements for subsequent chitin isolation. Nitrogen-based plasma removed 90% of proteins, while lactic acid ensured complete mineral removal, providing a near-zero-waste approach for chitin purification.	Green and zero-waste approach. Early-stage technology.	[25,26]
Crab (*Callinectes sapidus*)	Chitin, protein hydrolysate, and astaxanthin	Enzymatic hydrolysis (3% Alcalase/Bromelain). Followed by acetone extraction of astaxanthin (48 h, room temperature), acid demineralization (7% HCl, 1 h), and alkaline deproteinization (2% KOH, 2 h, 90 °C) for chitin recovery.	30% protein recovery (3% Alcalase, 120 min) Astaxanthin yield: 3.1 ± 0.4% (*w*/*w*) and astaxanthin content: 97.7 ± 14.3 μg/g residue Chitin recovered from enzymatically hydrolyzed residue.	Multi-product valorization. Multi-step process and solvent usage increase complexity.	[27]
*Jasus edwardsii* lobster (Liver)	Polyunsaturated fatty acids (PUFAs) enriched lipids	Supercritical fluid extraction: P: 35 MPa. CO2 flow rate: 0.434 kg/h, 50 °C for 4 h.	94% lipid extraction efficiency; PUFAs enriched to 31.3% of total lipids. DHA and EPA increased 7-fold. Heavy metals reduced by 0.5–27-fold compared with Soxhlet extraction.	Solvent-free and selective High capital cost	[6]
*Litopenaeus vannamei* hepatopancreas	Omega-3 and unsaturated fatty acid enrichment of shrimp oil	Solvent extraction method: Hexane and isopropanol, followed by purification (2–5 g of anhydrous sodium sulphate) and filtration. Extracted oil was enriched with omega-3 and unsaturated fatty acids using lipase hydrolysis.	Maximum enrichment achieved under optimized conditions Yielding DHA/EPA levels of 40.96%/22.61% and a 27.83% increase in total unsaturated fatty acids.	Nutrient enrichment achieved. Solvent use remains a limitation.	[28]
*Litopenaeus vannamei* cephalothorax	Cholesterol-reduced astaxanthin-rich shrimp oil	Silica gel column chromatography using sequential hexane/acetone elution (100:0 to 90:10) for cholesterol removal and astaxanthin fractionation. Followed by HPLC purification.	Removed cholesterol-rich fractions representing 26% of total components Astaxanthin-enriched fraction contained ~30% astaxanthin HPLC purification achieved 87% astaxanthin purity.	High purity. Not scalable and cost-intensive.	[29]
*Litopenaeus vannamei* shrimp (Head)	PUFA- and carotenoid-rich shrimp lipid extract	Ultrasound-assisted extraction (UAE) from lipid-containing solid residue (LSR) of shrimp cephalothorax using amplitudes of 60–100% under continuous or pulse mode (5–30 min).	Maximum lipid yield of 10–11 g/100 g. Carotenoid content of 8.6–8.8 mg/g lipid was achieved at 80% amplitude, prolonged ultrasonication increased lipid oxidation (PV, TBARS) and free fatty acid formation.	High yield. Oxidation risk with prolonged sonication.	[30]
Antarctic krill	Antarctic krill oil	Solvent extraction using Folch, acetone/ethanol (1:1), ethanol (95%), n-hexane/ethyl acetate (7:3), n-hexane/ethanol (10:1), and isopropanol (95%).	Ethanol (95%) achieved the highest glycerophospholipids (34.39%), EPA (20.83%), DHA (9.61%), and tocopherols (114.29 μg/g). Folch extraction yielded the highest astaxanthin content (283.49 μg/g).	Flexible solvent systems. Variability and solvent residue concerns.	[31]
Blue crab (*Portunus segnis*)	Carotenoprotein	Comparative extraction (enzymatic, maceration, Soxhlet) using solvents of varying polarity. Optimum extraction achieved by enzymatic pretreatment combined with hexane/isopropanol (50:50) maceration.	Hexane/isopropanol (50:50) maceration produced the highest carotenoid content. Enzymatic pretreatment significantly enhanced extraction selectivity toward astaxanthin. HR-ESI-MS identified 23 compounds.	Selective extraction. Solvent-intensive process.	[32]
Crustacean waste	Astaxanthin	Natural DES-assisted extraction using terpene-based natural deep eutectic solvents (menthol: myristic acid, 8:1) under optimized temperature and extraction time conditions.	Menthol: myristic acid (8:1) achieved astaxanthin yields comparable to Soxhlet extraction with acetone. For other biomasses, NADES extraction increased astaxanthin recovery by 3- to 657-fold compared with Soxhlet extraction.	Green and highly efficient. Solvent tuning required.	[33]
*Farfantepenaeus paulensis* shrimp (head, tail and shell)	Astaxanthin and omega-3 fatty acids	Supercritical fluid extraction: CO_2_/ethanol (15% wt. of ethanol), P:300 bar, 50 °C	15 wt.% ethanol produced the highest recoveries. 93.8% lipid recovery and 65.2% astaxanthin recovery. Lipid extraction yield increased by 136% compared with lower ethanol concentrations, with enhanced EPA and DHA recovery.	High recovery and selective co-extraction. High-pressure operation increases equipment cost.	[34]
*Penaeus vannamei* Boone	Astaxanthin	High-pressure extraction: Ethanol, liquid-to-solid ratio of 20 mL/g and 200 MPa for 5 min.	Higher astaxanthin recovery and antioxidant activity than conventional extraction. Ethanol (20 mL/g) was identified as the optimal solvent system	Improved recovery and bioactivity. Limited industrial scalability due to pressure requirements.	[35]
*Penaeus monodon* Shrimp (Head and shells)	Astaxanthin	Supercritical fluid extraction: 60 °C, P: 20 MPa. Solvent: 50% ethanol diluted in water.	Maximum carotenoid yield of 84.02 μg/g DW. 97.1% recovery was achieved using ethanol. Astaxanthin complex and free astaxanthin contents were 58.03 and 12.25 μg/g DW, respectively.	High recovery High equipment cost	[36]
Crab shells (*Portunus pelagicus*)	Vaterite-rich precipitated calcium carbonate	Gas–solid–liquid carbonation process	Recovered high-purity vaterite-phase PCC suitable for potential biomedical applications.	Sustainable mineral recovery. Process control required for phase stability.	[37]
Crab shells	Calcium-rich biochar	Pyrolysis	Calcium content ranged from 22.91 to 36.14%. Phosphorus removal efficiencies reached nearly 100% in phosphate solution and 63% in biogas effluent.	Waste valorization. Product variability.	[38]
Shrimp shell	Calcium	Calcination of fresh and dried shrimp shell waste (200–1000 °C) Followed by CaO-mediated hydroxyapatite (HAp) synthesis	Maximum calcium recovery of 71.47% at 1000 °C. Successfully produced HAp and carbonated HAp.	Simple process. High energy requirement.	[39]
Spiny lobster	Trypsin-like serine proteases (brachyurin-related enzymes)	Cloning and molecular characterization from digestive gland of *Panulirus argus.* Followed by sequence analysis and 3D comparative modeling.	Multiple trypsin isoforms identified. Suggesting at least three gene families encoding digestive trypsin.	High-resolution molecular insight.	[40]
Northern shrimp (*Pandalus borealis*)	Brachyurins/Serine protease	Purification from shrimp hepatopancreas using sequential chromatographic procedures. Followed by molecular cloning and enzymatic characterization.	Purified to homogeneity. Exhibited broad pH activity and stability profiles with distinct substrate specificity compared with mammalian cathepsins.	High specificity	[41]
Northern shrimp (*Pandalus eous*)	Cathepsin L-like cysteine protease	Crude extraction followed by hydroxyapatite chromatography. Superdex 75 gel filtration and Mono Q ion-exchange chromatography using DNP-Pro-Gln-Gly-Ile-Ala-Gly-Gln-d-Arg as activity substrate.	Purified to homogeneity (as evidenced by chromatographic and electrophoretic analysis). Exhibited broad pH stability and distinct substrate specificity compared to mammalian cathepsins.	High specificity	[42]
Spiny lobster (*Panulirus interruptus*)	Digestive proteases (isotrypsins and isochymotrypsins)	Analysis of gastric juice, midgut gland, and intestinal contents using specific substrates, inhibitors, and gel electrophoresis.	Multiple protease isoforms identified across tissues with similar composition in digestive compartments. Activity observed at acidic pH (~3) and inhibited by pepstatin A, indicating presence of acid proteases. Enzymes are characterized by biochemical variability and functional digestive roles.	Provides functional enzymatic profiling. Limited direct extraction application and mainly physiological characterization.	[43]
Wild and farmed shrimp	Digestive α-amylases	Midgut gland homogenization followed by enzymatic assays and zymogram (gel electrophoresis) characterization.	Multiple amylase isoforms detected across species (7–10 bands). Farmed *L. vannamei* showed ~3-fold higher activity than wild shrimp. Enzymes were active at pH 7–8 and 40–50 °C, with loss of stability above 55 °C; activity strongly modulated by inhibitors and metal ions.	Good comparative enzymatic profiling.	[44]

Chitin’s solubility is largely influenced by two major factors: degree of deacetylation (DDA) and degree of polymerization (DP). A higher DDA increases the number of free amino groups (–NH_2_), which can be protonated under acidic conditions, thereby enhancing intermolecular interactions with solvents and improving solubility, as observed in chitosan. This process is known as deacetylation. The deacetylation is mainly influenced by the alkali concentration, reaction temperature, treatment time, and the characteristics of chitin source. Higher alkali concentrations, elevated temperatures, and longer reaction times generally increase the degree of deacetylation. For example, Mittal et al. [18] prepared shrimp shell chitosan using 50% NaOH (1:40 *w*/*v*; chitin to solvent) under various conditions (110 or 130 °C for 2, 4, and 6 h). All the samples varied in the yield and DDA, and the chitosan prepared at 130 °C for 4 h showed the highest yield (73.11%), crystallinity index (19.75%), and 85.28% DDA. Generally, high temperatures and NaOH concentrations increased the DDA. Nevertheless, harsh conditions for deacetylation such as high temperature and longer time may depolymerize chitosan, thus liberating low MW chitooligomers, which is associated with the decrease in yield. Recent studies have also reported that chitosan-based materials continue to attract attention due to their tunable properties and environmental compatibility, further supporting their relevance in ongoing biomaterial research [45]. 

In addition to the DDA, DP also plays a critical role in solubility. A higher DP corresponds to longer polymer chains, increased crystallinity, and stronger chain entanglement, all of which reduce solubility. Conversely, lower DP, as observed in chitooligosaccharide (COS), leads to shorter chains with reduced intermolecular interactions, thereby facilitating dissolution in aqueous systems. COS are typically produced via enzymatic or non-enzymatic hydrolysis of chitin or chitosan, and the process conditions strongly influence its molecular weight and degree of polymerization (Table 1). Non-enzymatic hydrolysis of chitosan can be achieved through chemical and physical methods. Chemical approaches include acid hydrolysis (HCl and organic acids such as acetic, lactic, and formic acids) and oxidative methods using H_2_O_2_ or persulfate. During cleavage of β-1,4-glycosidic bonds, the resulting hydrolyzed chitosan comprises a mixture of N-acetylglucosamine and glucosamine units, with free aldehyde and hydroxyl groups formed at the cleavage sites. Hydrolysis rate is controlled by catalyst concentration, substrate concentration, temperature, reaction time, and the DDA of the starting chitosan [46].

Enzymatic hydrolysis of chitosan employs either specific enzymes (chitosanases) or non-specific enzymes including lipase, cellulase, pepsin, amylase, and carbohydrase. Although chitosanases offer high specificity, their high cost and limited availability restrict industrial-scale application, driving interest in non-specific alternatives. Non-specific enzymes hydrolyze chitosan through endo- or exo-action mechanisms on β-1,4-glycosidic bonds, producing COS of varying molecular weight and DP. Hydrolytic efficiency is strongly influenced by pH, temperature, enzyme-to-substrate ratio, and degree of deacetylation (DDA) of the chitosan substrate. Optimal hydrolysis generally occurs around pH 4–5, where reduced positive charge on the chitosan chain enhances enzyme accessibility to GlcN–GlcNAc and GlcNAc–GlcNAc linkages. At pH value above 5 leads to chitosan precipitation and reduced hydrolysis rates [46].

### 2.2. Protein and Bioactive Peptides

The crustacean processing industry generates substantial quantities of by-products that are abundant in high-quality proteins and essential amino acids [9,47]. Shrimp head contains 50–65% protein on a dry-weight basis and are considered a rich source of essential amino acids [48]. Similarly, lobster processing residues are also rich in protein, featuring an amino acid composition comparable to that of red meat, although they contain a higher proportion of non-protein nitrogen (10–40%) [49]. The protein content in lobster liver can reach up to 41% of its dry matter, while the head portion accounts for approximately 20% of the lobster’s total weight [50]. However, protein composition and recovery potential vary considerably depending on crustacean species, anatomical fraction, processing conditions, and techniques employed. Proteins can be recovered as concentrates or isolates by separating and purifying intact protein fractions from crustacean processing by-products. Another widely used approach is hydrolysis, in which peptide-bond cleaved to breaks large, folded proteins into smaller peptides and free amino acids [51]. Conventionally, hydrolysates have been produced using chemical processes, microbial fermentation, or commercial enzyme treatments. Chemical hydrolysis using strong acids or alkalis at high temperature is fast and inexpensive but non-selective, often degrading amino acids and offering little control over peptide size [52]. Microbial fermentation employs proteases secreted by microorganisms under mild conditions and can generate additional bioactive compounds, although it is slower and less reproducible [53]. In contrast, treatment with commercial proteases such as alcalase offers selective cleavage under mild, controllable conditions, allowing the degree of hydrolysis and thus peptide size and bioactivity to be reproducibly tuned while preserving nutritional quality [17]. This approach is therefore more suitable for producing hydrolysates with defined functional or bioactive properties.

Proteins can also be extracted from process waters generated during various industrial processes such as surimi production or the peeling and cooking of shellfish and krill [54]. For example, krill wash-water proteins have been recovered using sequential microfiltration (1.2 μm) and ultrafiltration (30 kDa) after washing in 0.5% sodium bicarbonate (pH 7) at 0–3 °C, producing a concentrated protein fraction [55]. These processing streams contain sarcoplasmic proteins and other soluble compounds but also significant amounts of functional myofibrillar proteins [56]. For example, Gautam et al. [17] reported the extraction of protein isolate from *Litopenaeus vannamei* shells through demineralization using HCl (1:20, room temperature) followed by alkaline solubilization with NaOH (1:30 at 60 °C), yielding a functional protein isolate from crustacean waste. Recovering these proteins not only helps minimize environmental pollutants but also adds value to crustacean processing waste. Recovery techniques include centrifugation, precipitation, membrane filtration (micro- or ultrafiltration), or their combinations [57,58].

Overall, enzyme-assisted hydrolysis is most suitable for producing high-value bioactive peptides because it provides greater control over product properties, whereas membrane filtration is more practical for recovering soluble proteins from dilute processing streams. Chemical hydrolysis remains economically attractive for bulk production but is less suitable when amino acid preservation and peptide specificity are priorities. Despite significant advances in protein recovery and hydrolysis technologies, challenges remain regarding process scalability, enzyme cost, membrane fouling, variability in raw material composition, and standardization of hydrolysate properties. Furthermore, comprehensive evaluation of techno-economic feasibility and environmental impacts is required before these approaches can be widely implemented at industrial scale. Therefore, industrial implementation should balance product value and quality against enzyme cost, membrane fouling, water consumption, and downstream purification requirements.

### 2.3. Lipids

Crustaceans are notable for their considerable amounts of omega-3 long-chain polyunsaturated fatty acids (PUFAs), especially eicosapentaenoic acid (EPA) and docosahexaenoic acid (DHA) [59]. Among marine-derived bioactive compounds, PUFAs have gained significant attention due to their well-documented health-promoting effects [60]. The lipid composition in crustacean processing residues varies significantly depending on the species, geographical origin, season, diet, and type of by-product. Albalat et al. [61] demonstrated that oil extracted from the head waste of Norway lobster (*Nephrops norvegicus*) contains higher levels of EPA (15.0%) and DHA (8.3%) within its neutral lipid fraction than krill oil, which contains 4.3% EPA and 2.3% DHA. Anatomical source also strongly influences lipid yield and composition. Raju et al. [62] reported that the cephalothorax and hepatopancreas from shrimp are valuable lipid sources rich in polyunsaturated fatty acids (PUFAs), yielding approximately 2.7% and 11.6%, respectively.

Lipid extraction from crustacean waste often involves a mixture of polar and non-polar solvents to maximize the recovery of both triglycerides and bioactive lipids such as omega-3 fatty acids and astaxanthin [32]. The basic principle of that extraction is “like dissolves like”; recovery is enhanced by matching solvent polarity to the targeted lipids or bioactives. For example, non-polar solvents (e.g., hexane) dissolve neutral lipids such as triglycerides, while the polar solvents (e.g., methanol or ethanol) disrupts lipid–protein complexes and solubilizes polar and amphiphilic lipids, including phospholipid-bound omega-3 fatty acids and the carotenoid astaxanthin. Solvent selection therefore influences both extraction yield and the composition of the recovered oil. Xie et al. [63] used sequential extraction with acetone, hexane, and ethanol, enabling selective recovery of krill oil fractions with distinct characteristics (total yield ~18.99%). The acetone fraction was enriched in minor bioactive compounds (astaxanthin, tocopherols, vitamin A, and cholesterol), whereas the ethanol fraction contained the highest levels of phospholipids (59.52%) and n-3 PUFAs (41.74%). Overall, ethanol-derived fractions exhibited the highest antioxidant capacity owing to their enrichment in phospholipids and n-3 PUFAs, whereas acetone and hexane preferentially recovered other lipid fractions, highlighting the importance of solvent selection in tailoring lipid functionality. 

In addition to the interspecies variation, different organs within the same species yield oils with different properties. Although the cephalothorax is commonly used for oil extraction, isolating the hepatopancreas produces higher oil yield with enhanced properties. For example, a comparative analysis of shrimp oil extracted from the cephalothorax (CPO) and hepatopancreas (HPO) of Pacific white shrimp revealed significant differences in yield and nutritional quality [62]. The hepatopancreas produced a substantially higher oil yield (~11%) than the cephalothorax (~3%) and was richer in astaxanthin and PUFAs, particularly DHA, while containing lower cholesterol levels. In contrast, CPO contained a higher proportion of saturated fatty acids (SFAs). Nutritional indices, including the PUFA/SFA and hypocholesterolemic/hypercholesterolemic ratios, further supported the superior nutritional quality of HPO [64]. However, oxidation analysis indicated differences in stability, with CPO exhibiting higher primary oxidation products, whereas HPO showed greater secondary oxidation compounds [62]. Seafood lipids may also contain high levels of SFAs and cholesterol, which are associated with several health concerns. Therefore, several methods such as chromatography, winterization, ultrafiltration, and enzymatic hydrolysis have been used to reduce the SFA content [28]. As a recent example, Gautam et al. [28] reported that lipase-assisted hydrolysis significantly increased DHA (40.96%) and EPA (22.61%), along with total unsaturated fatty acids (27.83%). This enrichment of unsaturated fatty acids consequently led to a relative decrease in the proportion of SFAs in the modified oil.

In addition to fatty acids, cholesterol is another important component of crustacean lipids. In Pacific white shrimp by-products, including the cephalothorax, pleopods, shells, and tails, cholesterol levels of approximately 65 ± 1 mg/g have been reported, whereas edible shrimp contains over 100 mg cholesterol per 100 g tissue [65,66]. Owing to the relatively high cholesterol content of shrimp lipids, various purification strategies have been developed to enhance their nutritional quality. β-Cyclodextrin (β-CD), saponin-assisted treatment, ethanol fractionation, and chromatographic purification have all demonstrated effective cholesterol removal (up to ~98%) while simultaneously enriching astaxanthin, EPA, DHA, and other unsaturated fatty acids, resulting in improved nutritional quality and oxidative stability [29,67,68]. Chromatographic fractionation further enabled selective separation of cholesterol and astaxanthin, producing purified lipid fractions with enhanced functional properties [68]. Although naturally occurring antioxidants in crustaceans contribute to oxidative stability, effective preservation strategies are essential for broader industrial utilization [69,70]. Gulzar et al. [71] demonstrated that encapsulating shrimp oil in chitosan tripolyphosphate nanoparticles significantly improved oxidative stability and the retention of bioactive compounds, including PUFAs and astaxanthin, during storage. This enhanced stability was attributed to the protective barrier provided by the nanoparticles and the inherent antioxidant properties of chitosan.

Overall, solvent extraction remains effective for high lipid recovery, with food-grade ethanol showing promise for obtaining phospholipid- and omega-3-rich fractions. Enzymatic enrichment, cholesterol removal, and encapsulation provide additional nutritional and stability benefits but increase processing complexity and cost. Therefore, industrial implementation should balance extraction selectivity, solvent recovery, oxidative stability, and the value of the intended product.

### 2.4. Carotenoids

Carotenoids are naturally occurring lipophilic pigments widely distributed in marine organisms [72]. As crustaceans cannot synthesize carotenoids on their own from basic metabolic precursors, these pigments are accumulated through the diet and subsequently metabolically modified, resulting in variations in carotenoid composition among species and by-products. Crustacean processing residues are particularly rich in carotenoids, predominantly astaxanthin, together with minor carotenoids such as β-carotene, lutein, zeaxanthin, canthaxanthin, and β-cryptoxanthin, which contribute to their antioxidant and nutritional properties [72,73,74]. Astaxanthin concentrations vary considerably among crustacean species (24-199 µg/g), depending on dietary carotenoid intake, species-specific metabolism, environmental conditions, and tissue composition [75,76,77]. El Boumlasy et al. [78] further demonstrated that shrimp by-products contain multiple astaxanthin derivatives, including free, monoesterified, and diesterified forms, with shrimp heads exhibiting the highest astaxanthin content among the evaluated by-products. Several studies have shown that solvent selection strongly influences astaxanthin recovery from crustacean by-products. Acetone consistently produced the highest extraction yields from shrimp waste, whereas substantially lower recoveries were obtained from crab exoskeletons because astaxanthin is more tightly bound within the highly calcified chitin–protein matrix of crab shells [78,79]. Response-surface optimization further confirmed that organic solvents provided higher carotenoid recovery than vegetable oils and that shrimp waste yielded more carotenoids than crab waste under optimized conditions [79]. These findings highlight the importance of selecting appropriate extraction systems according to both solvent properties and the structural characteristics of the raw material. From an industrial perspective, the greater extraction efficiency of organic solvents must be balanced against solvent-recovery and residual-solvent requirements, whereas edible oils provide a formulation-compatible alternative but generally achieve lower carotenoid recovery.

Beyond extraction efficiency, the stability and antioxidant functionality of astaxanthin are also critical for its practical applications. Pu et al. [80] investigated astaxanthin extracted from shrimp (*Litopenaeus setiferus*) by-products using flaxseed oil and demonstrated its effectiveness in enhancing oxidative stability. The astaxanthin-enriched oil exhibited a lower lipid oxidation rate compared to control oil, particularly at elevated temperatures (40–60 °C), confirming its role as a potent natural antioxidant. Nevertheless, astaxanthin degradation followed first-order kinetics and increased markedly at 50–60 °C compared with 30–40 °C. Therefore, although astaxanthin can improve lipid oxidative stability, temperature control during extraction, processing, and storage remains essential for preserving the pigment.

### 2.5. Calcium Carbonate

Calcium carbonate (CaCO_3_) represents another valuable compound recovered during the pretreatment of marine shell wastes and is widely utilized across various industrial sectors. Crab shells have been identified as a rich source of calcium carbonate, from which high-purity CaCO_3_ can be efficiently extracted through calcination and precipitation processes, often yielding a high proportion of vaterite crystalline phase (~98%), which is advantageous due to its enhanced reactivity and bioavailability [81]. Similarly, Bayuseno et al. [37] reported that crab shells (*Portunus pelagicus*), containing approximately 70 wt.% calcium carbonate, can be effectively utilized for the synthesis of precipitated calcium carbonate (PCC) via a gas–solid–liquid carbonation process. The resulting PCC predominantly exhibited the vaterite crystalline phase, which is highly desirable for biomedical applications due to its enhanced bioactivity and solubility, highlighting the potential of crab shell waste as a low-cost source of functional biomaterials. Through thermal processing, the calcium content in these wastes can be transformed into calcium oxide [82]. In addition, crab shell-derived calcium-rich biochar produced via pyrolysis can exist in either calcite- or lime-based forms depending on the pyrolysis temperature, with calcite (CaCO_3_) predominating at temperatures ≤ 600 °C, a transitional mixture of CaCO_3_ and CaO forming between 600 and 700 °C, and lime (CaO) becoming dominant at temperatures ≥ 700 °C [38]. Shrimp shells have also been highlighted as a rich source of both calcium and phosphorus [83]. Zakaria et al. [39] performed calcination at elevated temperatures (up to 1000 °C) and noticed that the calcium recovery efficiency increased significantly from 29.51% to 71.47%, indicating effective conversion of shell waste into calcium-rich materials. Furthermore, the obtained calcium oxide was successfully utilized for the synthesis of hydroxyapatite, including both pure and carbonate-substituted forms.

Overall, the most appropriate conversion pathway depends on the intended product: carbonation and precipitation are suitable for producing high-purity PCC, pyrolysis can generate calcium-rich biochar for environmental applications, and high-temperature calcination provides CaO for hydroxyapatite synthesis. However, the greater energy demand of high-temperature processing must be considered when evaluating its economic and environmental feasibility at an industrial scale.

### 2.6. Enzymes and Enzyme Inhibitors

Crustacean internal organs, particularly the hepatopancreas and associated visceral tissues, contain a wide variety of enzymes with significant biological and biotechnological relevance (Table 1) [84]. Many of these enzymes exhibit cold-adapted properties (active between 0 and 30 °C but unstable at temperatures higher than 50 °C), especially those derived from species inhabiting cold and temperate marine environments [85]. In crustaceans, digestive proteases are predominantly localized in the hepatopancreas, which functions as the primary organ for digestion and enzyme secretion. Among crustacean proteases, serine endopeptidases, collectively referred to as brachyurins, are the most extensively studied and represent the major class of digestive proteases in decapod crustaceans [86]. Brachyurins are structurally and functionally related to vertebrate pancreatic proteases and are responsible for the efficient degradation of dietary proteins. Brachyurins include enzymes with broad trypsin-, chymotrypsin-, elastase-, and collagenase-like activities, as well as more substrate-specific trypsin-like proteases [40,86,87]. Recently, trypsin from harpiosquillid mantis shrimp was successfully purified using ammonium sulfate precipitation followed by affinity chromatography (soybean trypsin inhibitor–Sepharose 4B), resulting in a 30.4-fold purification with a yield of 14.5% and a molecular weight of ~23 kDa, highlighting the effectiveness of combined precipitation–chromatographic approaches [88]. In addition, Northern shrimp species such as *Pandalus borealis* and *Pandalus eous* were also used to isolate cathepsin L-like proteinase and gelatinolytic proteases (A1, A2, and B). Among these, proteases A2 and B exhibited collagenolytic activity, effectively degrading native type I collagen, and were identified as serine proteases based on their strong inhibition by PMSF and antipain [41,42]. Further insights into crustacean serine proteases (brachyurins, EC 3.4.21.32) have been obtained from studies on the spiny lobster *Panulirus argus*. Multiple trypsin-like proteases were cloned from the digestive gland, revealing that at least three gene families encode trypsin in this species. Structural modeling suggested differences in substrate and inhibitor interactions among these enzymes. While most were typical trypsin, one variant could not be classified within known brachyurin groups due to key amino acid substitutions near the active site, which likely modify substrate binding and specificity. This finding indicates that substrate specificity within the brachyurin family may be broader than previously understood [43,89]. Similar protease compositions were observed in gastric juice, midgut gland, and intestinal contents, indicating a coordinated digestive system (Table 1). Using specific substrates, inhibitors, and electrophoretic techniques, multiple isoforms of trypsin and chymotrypsin were identified. Protease activity was detected even at acidic pH (around pH 3), with inhibition by pepstatin A suggesting the presence of aspartic proteases alongside serine proteases. Accurate identification and characterization of crustacean-derived enzymes require the application of complementary analytical techniques. As summarized in Table 1, enzyme studies commonly employ substrate-specific activity assays, electrophoretic techniques (e.g., SDS-PAGE and zymography), chromatography-based purification methods, and molecular approaches such as gene cloning and sequence analysis. In addition, advanced analytical tools including HPLC, mass spectrometry, and protein structural modelling have been utilized to evaluate enzyme purity, molecular characteristics, and catalytic properties. The selection of appropriate analytical techniques is essential for confirming enzyme identity, functionality, and potential industrial applications.

In addition to proteases, lipases, phospholipases, and carbohydrases are also obtained from crustacean waste, especially from the hepatopancreas, which serves as the principal digestive and secretory organ (Table 1). Phospholipase activity has been reported in the hepatopancreas and digestive glands of several crustacean species, including crabs such as *Carcinus mediterraneus* [90]. Amylase activity has been widely reported in the digestive system of crustaceans, with the hepatopancreas and midgut gland serving as the primary sites of enzyme synthesis and secretion. An isolation and characterization study conducted by Maalej et al. [91] identified amylases in the viscera of the blue crab (*Portunus segnis*) using a multi-step approach involving ultrafiltration, followed by Sephadex G-100 gel filtration and Mono Q anion-exchange chromatography, achieving a high purification fold (424.02) with 27.8% recovery. The purified enzyme (~45 kDa) exhibited optimal activity at 50 °C, broad pH stability, and high tolerance to surfactants, indicating strong industrial applicability. 

In addition to the digestive enzymes, crustaceans possess an efficient chitinolytic enzyme system that plays a crucial role in molting, digestion, and exoskeleton remolting. Chitin degradation in crustaceans is primarily mediated by the synergistic action of endo-type chitinases (EC 3.2.1.14) and exo-type β-N-acetyl-hexosaminidases (EC 3.2.1.52). Chitinases cleave internal β-(1,4) glycosidic bonds within chitin, producing N-acetyl-chitooligosaccharide, which are subsequently hydrolyzed into N-acetyl-D-glucosamine monomers by β-N-acetyl-hexosaminidases [92]. These enzymes are essential for the complete depolymerization of chitin during digestive and physiological processes in crustaceans. They are predominantly localized in the hepatopancreas and digestive tract, where they facilitate the breakdown of chitinous material derived from dietary sources as well as endogenous chitin turnover associated with molting. Significant chitinase and β-N-acetyl-hexosaminidase activities have been reported in the viscera and digestive glands of several crustacean species, including crabs and shrimps, indicating their strong chitin-degrading capability. In addition to chitinolytic enzymes, chitosan-degrading enzymes (chitosanases, EC 3.2.1.132) have also been identified in crustaceans. Although chitosanases are more commonly associated with microorganisms, their occurrence in crustacean viscera has been documented, for example, in the hepatopancreas of the blue crab (*Portunus segnis*), red king crab (*Paralithodes camtschaticus*), and fiddler crab (*Uca pugilator*) [93,94,95]. These enzymes catalyze the hydrolysis of β-(1,4) glycosidic bonds in chitosan, leading to the formation of low-molecular-weight chitosan oligomers.

Transglutaminases are calcium-dependent acyltransferases that catalyze covalent cross-linking between proteins. Iijima et al. [96] identified cuticular proteins involved in transglutaminase-dependent cross-linking in the horseshoe crab *Limulus polyphemus*. However, this study demonstrated the physiological function of transglutaminase rather than its recovery from hemolymph or processing by-products. Therefore, evidence supporting the industrial extraction of transglutaminase from crustacean waste remains limited.

In addition to enzyme recovery, crustacean by-products are also recognized as valuable sources of natural enzyme inhibitors. Bioactive compounds such as protein hydrolysates, peptides, chitin, chitosan, and their derivatives have demonstrated the ability to inhibit key metabolic enzymes. These inhibitors primarily target digestive enzymes such as α-amylase, α-glucosidase, and pancreatic lipase, as well as enzymes involved in blood pressure regulation, including angiotensin-converting enzyme (ACE) and renin [57,97,98]. These findings support potential food and nutraceutical applications, although activity confirmation in biological systems and regulatory evaluation is required before commercial use.

## 3. Emerging Technologies Used in the Extraction of Bioactive Compounds from Crustaceans

The increasing demand for sustainable and efficient extraction methods has led to the exploration of innovative non-thermal technologies for recovering bioactive compounds and other valuable components from crustacean by-products [99]. These methods are gaining significant attention due to their ability to enhance product quality while minimizing energy consumption and environmental impact (Table 1). Among the various non-thermal techniques, supercritical fluid extraction, high-pressure processing, pulsed electric fields, and ultrasound have emerged as promising alternatives for improving the recovery efficiency of bioactive substances from crustacean waste.

### 3.1. Deep Eutectic Solvents (DES)

Considering the toxicity and environmental impacts of traditional chemicals, food scientists have shown increasing interest in deep eutectic solvents (DESs). They are a novel class of green solvents formed by combining hydrogen bond donors (HBDs) and acceptors (HBAs) to produce a eutectic mixture with a melting point lower than that of its individual components, generally exhibiting low volatility and tunable physicochemical properties. However, their toxicity and biodegradability depend on their composition and should be evaluated individually. Due to their ability to reduce reliance on strong acids and alkalis while selectively dissolving biopolymers, DESs have gained significant attention as sustainable alternatives to conventional solvents for biomass valorization, including crustacean waste. The extraction efficiency is strongly influenced by solvent composition, as different HBDs (organic acids, polyols) and HBAs (e.g., choline chloride) alter hydrogen-bond strength, viscosity, and acidity, thereby affecting mass transfer and polymer stability. For instance, DESs based on choline chloride combined with different organic acids have been evaluated for extracting chitin from lobster shells, where the purity and molecular weight of the extracted chitin were found to depend strongly on the type of acid and processing temperature. DESs extract chitin by forming extensive hydrogen-bonding networks that disrupt the interactions between chitin, proteins, and minerals. The acidic components of DES dissolve calcium carbonate through protonation, while simultaneously weakening protein–chitin interactions and promoting protein solubilization. For example, among the systems studied, choline chloride–malonic acid yielded chitin with the highest purity (93%). Moreover, molecular weight variation was observed, with malonic acid producing chitin of 312 kDa at 50 °C and 199 kDa at 100 °C, while malic acid at 100 °C yielded significantly lower molecular weight (91 kDa) [19]. Rodrigues et al. [33] first explored the application of a natural DES for the extraction of astaxanthin from crustacean waste. Terpene-based natural DES, particularly menthol: myristic acid (8:1), achieved extraction yields comparable to conventional Soxhlet extraction, while significantly enhancing astaxanthin recovery (3- to 657-fold) from various biomasses, including shrimp shells. Subsequently, Rodrigues et al. [20] demonstrated the effectiveness of DES for eco-friendly chitin extraction. To evaluate the environmental effects of DES, choline chloride (ChCl)/organic acid-based DESs were investigated for their phytotoxicity using wheat seed assays. Among the systems tested, ChCl/lactic acid DES exhibited the lowest phytotoxicity (EC_50_ ≥ 1.6 mg/mL) and enabled the extraction of high-purity chitin (~98%) from brown crab shells at 130 °C, with a processing time more than eight times faster than conventional methods. Similarly, Ma et al. [21] reported the extraction of chitin from snow crab (*Chionoecetes opilio*) shells using a DES composed of triethylbenzylammonium chloride (TEBAC) and lactic acid. The system effectively removed both proteins and minerals, achieving high deproteinization (95.51%) and decalcification (96.41%) efficiencies, with a chitin purity of 91.15% and a yield of 21.31%. Huang et al. [22] demonstrated a green extraction approach using a choline chloride–malic acid-based natural deep eutectic solvent, assisted by microwave irradiation, for chitin recovery from shrimp shells. The system efficiently removed minerals and proteins and produced chitin with a relative crystallinity of 71%. Furthermore, Zhu et al. [100] investigated the influence of different polyols in DES systems (ethylene glycol, glycerol, xylitol, and sorbitol) for chitin extraction from lobster shells. The study revealed that increasing hydroxyl groups enhanced hydrogen bonding but also increased viscosity, affecting extraction efficiency. Among the tested systems, choline chloride–lactic acid/glycerol (CCLaGly) demonstrated superior performance, yielding high-quality chitin with a purity of 94.76 ± 0.33%, crystallinity of 78.78%, and molecular weight of 655 kDa, comparable to chemically extracted chitin. Recently, Huang et al. [101] introduced a hydrophobic deep eutectic solvent (HDES) as an advanced approach to chitin extraction. A system composed of procaine, *L*-menthol, and acetic acid achieved efficient extraction under ambient conditions, with high demineralization (98.35%) and deproteinization (88.77%) efficiencies. The HDES preserved the native α-chitin structure, avoiding depolymerization and deacetylation typically associated with harsh chemical treatments. Notably, its intrinsic hydrophobicity enabled effective phase separation, allowing easy solvent recovery and reuse over multiple cycles with minimal loss in performance.

Overall, acidic DESs can produce high-purity chitin but often require elevated temperatures, whereas hydrophobic DESs offer ambient-temperature processing and easier solvent recovery. Nevertheless, solvent toxicity, cost, viscosity, recyclability, residual contamination, and scale-up feasibility require further evaluation before industrial implementation.

### 3.2. Supercritical Fluid Extraction

**Figure 2:**
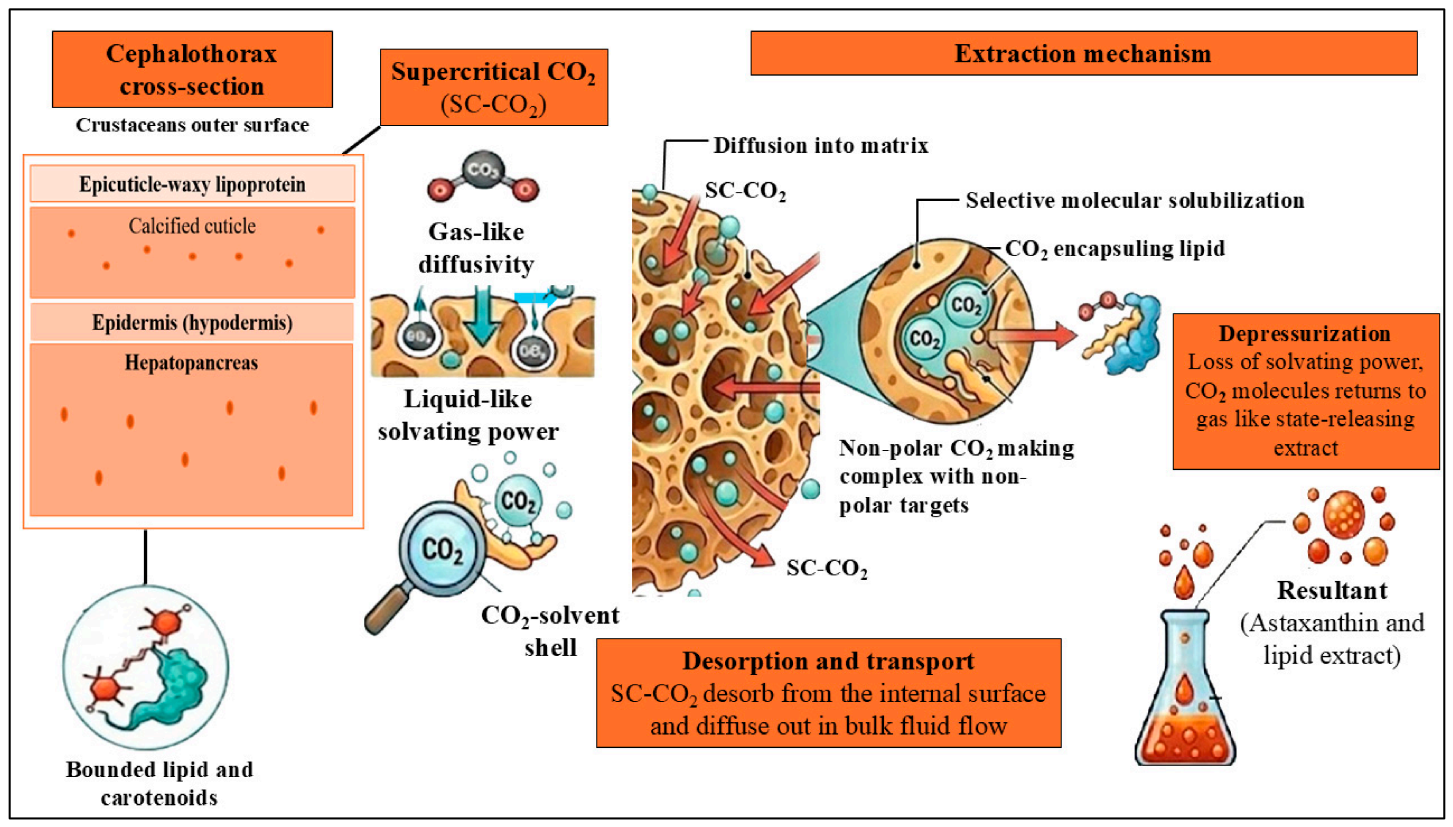
Schematic illustration of mechanism of supercritical fluid extraction (SFE) applied to crustacean waste.

Supercritical fluid extraction (SFE) is used to isolate specific compounds from various matrices, whether solid or liquid, by utilizing a supercritical fluid (SF). The principle of SFE is based on the use of a fluid above its critical temperature and pressure, where it exhibits both gas-like diffusivity and liquid-like solvating power (Figure 2). In this state, the supercritical fluid can penetrate the sample matrix like a gas and dissolve target compounds like a liquid. The solvating strength of the fluid can be precisely tuned by adjusting pressure and temperature, enabling selective extraction of specific compounds. Furthermore, rapid depressurization after extraction allows easy separation of the solvent from the extracted compounds, leaving no solvent residues [102]. SFs such as carbon dioxide (CO_2_) exhibit unique properties that blend the characteristics of both gases and liquids, including low viscosity, high diffusivity, and minimal surface tension [103,104]. These properties make them ideal for efficient extraction processes. While a range of supercritical fluids can be employed, CO_2_ stands out as the preferred solvent in the food industry due to its inert, non-toxic, non-flammable nature and cost-effectiveness. Supercritical CO_2_ (SC-CO_2_) extraction operates at relatively low temperatures (critical conditions at 30.9 °C and 73.8 bar), making it particularly suitable for heat-sensitive compounds such as carotenoids and lipids [105,106].

Application of SFE was demonstrated by Sánchez-Camargo et al. [34] for the extraction of lipids and astaxanthin from freeze-dried *Farfantepenaeus paulensis* by-products. At 300 bar and 50 °C, with a fixed solvent-to-feed ratio, increasing the ethanol concentration from 5% to 15% substantially improved lipid and astaxanthin recoveries to 93.8% and 65.2%, respectively. The extraction efficiency was optimized by adjusting parameters such as CO_2_ flow rate, moisture content, temperature, pressure, and co-solvent addition (hexane–isopropanol or sunflower oil). Optimal conditions were identified at a CO_2_ flow rate of 13.3 g/min, moisture content of ~11%, and high pressure (300 bar), which significantly enhanced CO_2_ density and carotenoid recovery, particularly astaxanthin. *Penaeus paulensis* waste (freeze-dried heads, shells, and tails) contained high protein (49% dry weight) and ash (27% dry weight) contents, with low lipid levels (4.9% dry weight), although the lipid fraction was rich in valuable polyunsaturated fatty acids such as EPA and DHA. The study compared traditional solvent extraction (TSE), SFE, and SFE with ethanol as a co-solvent for astaxanthin recovery. While SFE alone showed the lowest extraction efficiency, the addition of 10% (*w*/*w*) ethanol significantly enhanced astaxanthin recovery to 57.9%, approaching acetone extraction (63.3%). However, the highest carotenoid yield (53 mg/kg waste) was achieved using a 60% (*v*/*v*) n-hexane: isopropanol mixture [107].

Taken together, these studies highlight the central trade-off of SC-CO_2_ for crustacean carotenoids. Astaxanthin occurs in free and esterified forms, and its recovery using SC-CO_2_ alone can be limited by pigment polarity and interactions with the shell matrix. Consequently, SFE without a co-solvent gave the lowest recovery, and only the addition of ethanol (10–15%) raised astaxanthin recovery (57.9–65.2%) to a level approaching conventional acetone extraction (63.3%), while the highest absolute carotenoid yield was still obtained with a non-polar n-hexane:isopropanol mixture. Thus, although SFE offers a green, solvent-residue-free process based on GRAS, recyclable CO_2_ under mild temperatures that protect thermolabile carotenoids and polyunsaturated fatty acids, its intrinsically non-polar character necessitates organic co-solvents for polar pigments, which partly offsets its environmental advantage. In addition, the high capital and operating costs of high-pressure equipment and the limited processing throughput remain key barriers to industrial-scale adoption. A meaningful comparison of SFE against conventional solvent extraction therefore requires not only yield but also quantitative greenness and techno-economic indicators (e.g., solvent consumption, energy demand, and process mass intensity). Overall, SC-CO_2_ is particularly effective for extracting non-polar and slightly polar compounds, particularly those of medium molecular weight, and can maintain a non-oxidizing environment, thereby preventing the degradation of sensitive compounds during extraction [108].

### 3.3. High-Pressure Extraction or High-Pressure-Assisted Extraction (HPAE)

**Figure 3:**
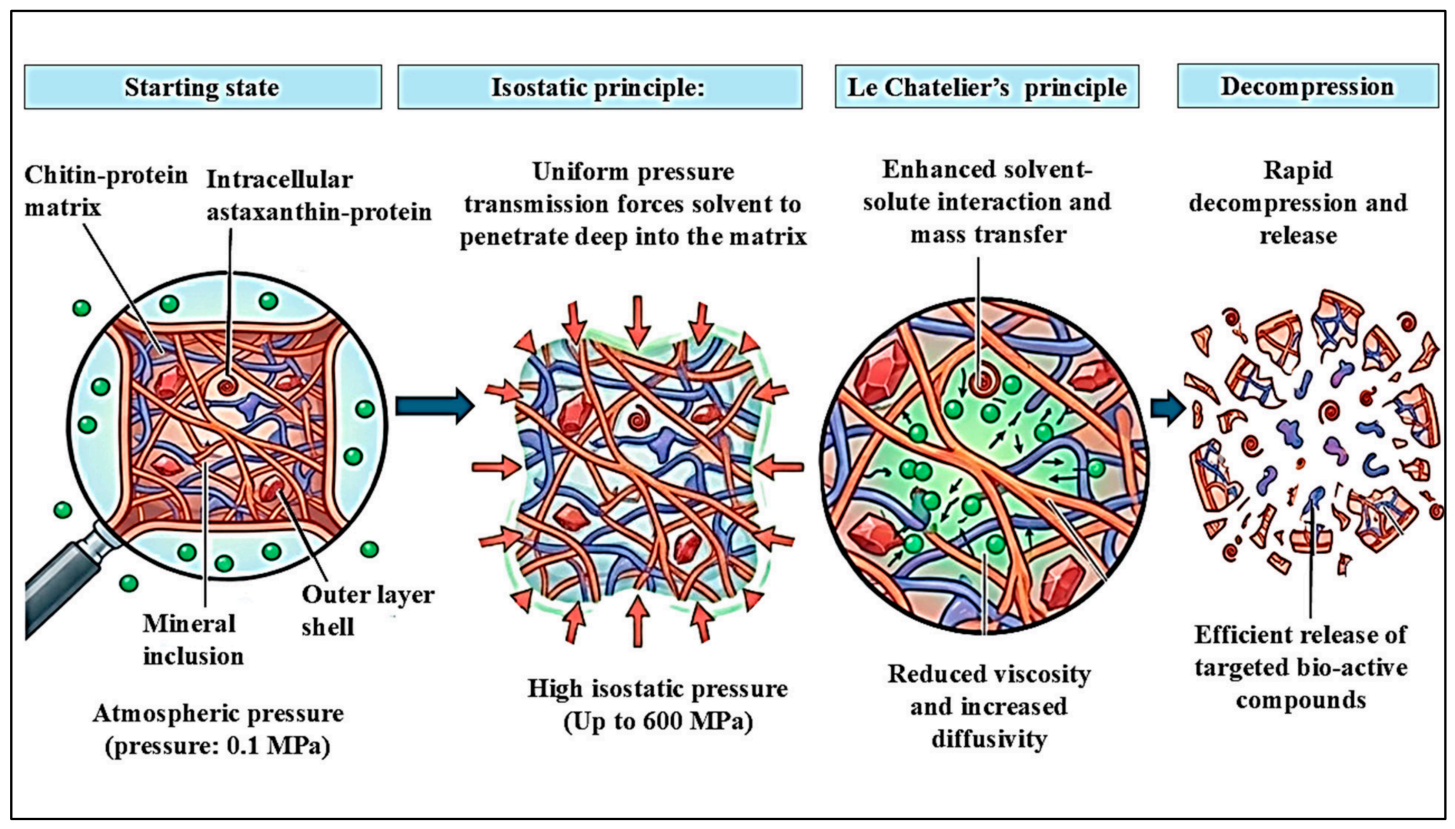
Schematic illustration of mechanisms of high-pressure extraction or high-pressure-assisted extraction (HPAE) from crustacean waste.

High-pressure extraction (HPE), or high-pressure-assisted extraction (HPAE), also known as high hydrostatic pressure extraction (HHPE), is a non-thermal extraction technique that uses high pressure (100–600 MPa) [109]. The principle of HPE is based on the uniform transmission of pressure (isostatic principle). The pressure allows the solvent to penetrate deeply into the sample matrix and disrupt cell walls and membranes, facilitating the release of intracellular compounds. According to Le Chatelier’s principle, pressure favors reactions and interactions that involve a decrease in volume, thereby enhancing solute–solvent interactions and mass transfer (Figure 3) [110]. Additionally, high pressure can enhance solvent penetration and mass transfer, thereby improving extraction efficiency. Rapid decompression further contributes to cell rupture, promoting the release of target bioactive compounds. Operating HPE at ambient or refrigerated temperature during the process of HPE minimizes thermal degradation and preserves the integrity of heat-sensitive bioactives. From the shells of *Litopenaeus vannamei*, Li et al. [35] recovered 71.1 μg/g astaxanthin using ethanol as the solvent at a liquid-to-solid ratio of 20 mL/g and 200 MPa for 5 min. Similarly, a carotenoid concentration of 68.26 μg/mL and an astaxanthin yield of 59.9744 μg/g dry weight were obtained from *Penaeus monodon* using an acetone–methanol mixture (7:3, *v*/*v*) at 210 MPa for 10 min [111]. Despite these advantages, HPAE presents several limitations that may restrict its industrial-scale application. The technique requires specialized high-pressure equipment, resulting in high capital investment and operational costs. In addition, energy consumption associated with pressurization and depressurization cycles can be considerable. The process is generally operated in batch or semi-batch mode, which limits continuous large-scale production. Moreover, extraction efficiency is highly dependent on matrix characteristics and process optimization, making standardization across different crustacean wastes challenging. These factors collectively limit the widespread industrial adoption of HPAE despite its promising laboratory-scale performance. Therefore, HPAE may be most suitable for rapidly recovering high-value, heat-sensitive compounds for which improved product quality can justify the additional equipment and processing costs.

### 3.4. Ultrasound-Assisted Extraction (UAE)

**Figure 4:**
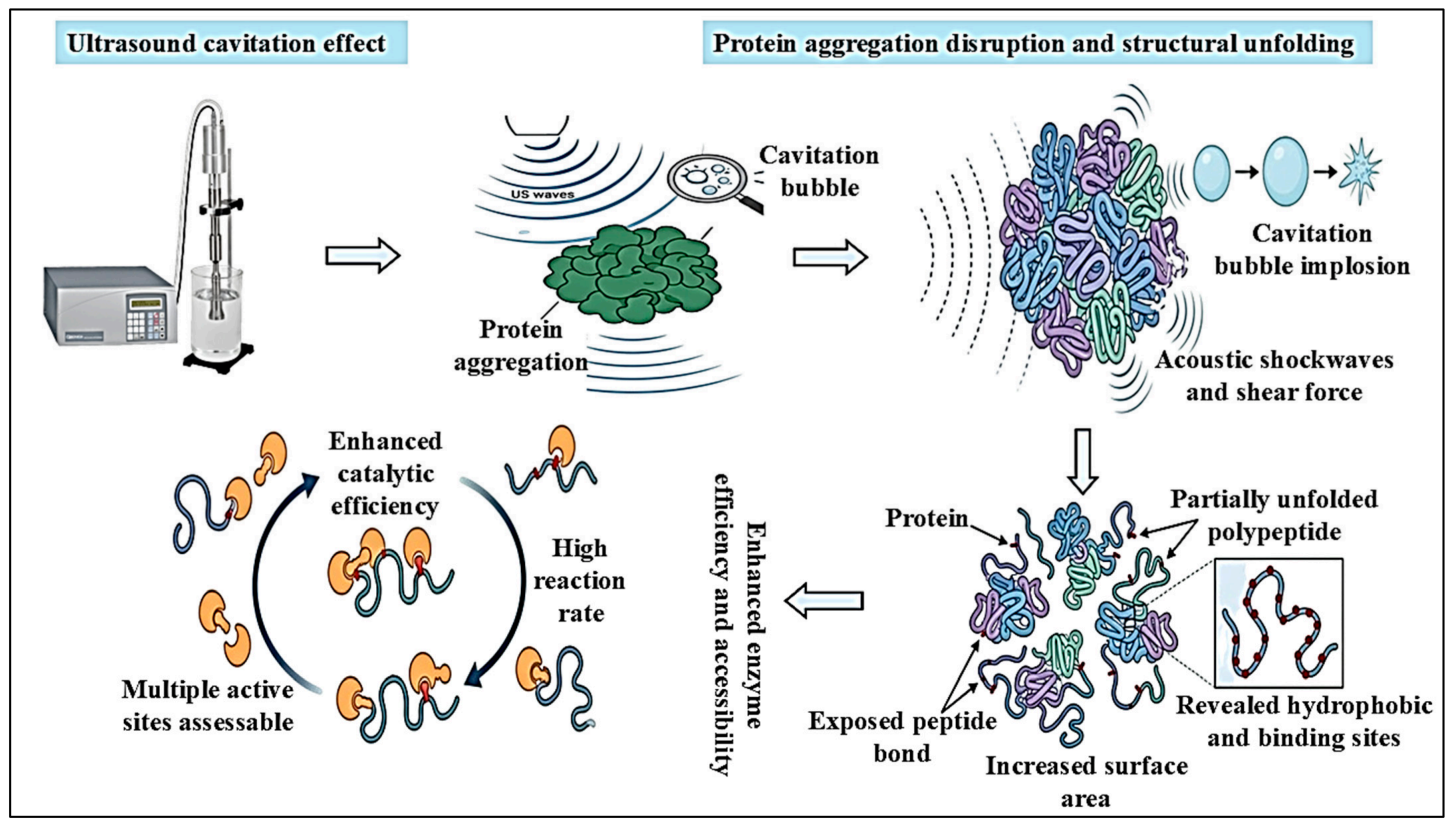
Schematic illustrations of cavitation effect and ultrasound-assisted enzymatic hydrolysis of protein extracted from crustacean waste.

Ultrasound is an environmentally sustainable approach in modern food processing [112]. The principle of UAE is based on the propagation of high-frequency sound waves (typically 20 kHz–100 MHz) through a medium, producing alternating compression and rarefaction cycles that drive the process. The key mechanism underlying ultrasound-assisted processes in liquid systems involves acoustic cavitation (Figure 4), which refers to the formation, growth, and implosive collapse of gas microbubbles. These cavitation events originate from the pressure fluctuations of the sound waves and lead to intense, localized energy release, enhancing mass transfer and cell disruption. These rapid events generate localized high temperatures and pressures, as well as intense shear forces, which contribute to the breakdown of cellular structures [113]. UAE has shown considerable promise in extracting bioactive compounds from both plant and animal matrices, and offers several advantages over conventional methods, including reduced solvent usage, faster processing, better reproducibility, easier handling, and increased purity of the final extracts. Gautam et al. [17] applied ultrasonication before enzymatic hydrolysis of a protein isolate prepared from the shells of *Litopenaeus vannamei*. Ultrasonication of the protein isolate at 60% amplitude for 15 min prior to enzymatic hydrolysis resulted in lower enzyme consumption. At optimized enzyme concentrations without ultrasonication, the degree of hydrolysis (DH) was reported as 22.15% for 2% alcalase and 13.75% for 3% papain; however, following ultrasonication pretreatment, DH significantly increased to 31.12% for alcalase and 42.94% for papain at the same enzyme concentrations. This improvement can be attributed to ultrasound-induced structural modification of proteins, where cavitation disrupts protein aggregates, increases surface area, and exposes more peptide bonds, thereby enhancing enzyme accessibility and catalytic efficiency (Figure 4). Kjartansson et al. [23] applied ultrasound-assisted demineralization and deproteinization using 0.25 M HCl and 0.25 M NaOH, respectively, to recover chitin from the shells of North Atlantic shrimp (*Pandalus borealis*). Chemical deproteinization reduced the protein content from 39.8% to 10.6% without sonication, while sonication for 1 and 4 h further reduced it to 8.3% and 7.3%, respectively. Additionally, ultrasound reduced the crystallinity index of chitin (from 87.6% to ~79%), indicating partial structural loosening, which may facilitate downstream processing such as deacetylation. However, sonication did not improve demineralization and reduced the chitin yield from 16.5% to 11.4%, which was attributed to the solubilization of depolymerized fragments into the washing medium. These findings suggest that while ultrasound is highly effective in improving protein removal and modifying structural properties, its application must be optimized to balance purity and yield. Similarly, Xing et al. [114] demonstrated that combining low-intensity ultrasound (0.167 W/cm^2^) with *Lacticaseibacillus paracasei* fermentation significantly enhanced chitin extraction from crab shells. Intermittent ultrasonication (10 min at 8 h intervals) stimulated bacterial growth and activity, leading to improved decalcification and deproteinization by 16.72% and 33.45%, respectively, while reducing the overall fermentation time to 48 h. The improvement is attributed to ultrasound-induced enhancement of mass transfer and cell permeability, which facilitates better interaction between microorganisms and the shell matrix. Structural analyses confirmed efficient removal of impurities without altering the α-chitin conformation, indicating that ultrasound-assisted fermentation can provide a promising alternative that reduces reliance on conventional chemical extraction. Overall, UAE is particularly promising as a pretreatment for enzymatic hydrolysis and biological chitin extraction; however, industrial implementation requires optimization of energy intensity, treatment duration, reactor geometry, temperature control, and product yield.

Investigations have highlighted the efficacy of UAE in recovering lipids and carotenoids from crustacean by-products, primarily due to the enhanced extraction performance provided by cavitation effects. Sinthusamran et al. [30] reported that ultrasound-assisted extraction at 80% amplitude yielded the highest lipid recovery (10–11 g/100 g) along with carotenoid content of about 8.6–8.8 mg/g lipid from lipid-containing solid residues generated after protein hydrolysis of Pacific white shrimp cephalothorax. However, it is important to note that, under certain conditions, ultrasound may also contribute to undesirable changes such as lipid oxidation or hydrolysis. For example, increased peroxide value (PV) and thiobarbituric acid reactive substances (TBARS), along with elevated free fatty acid (FFA) content, were observed with increasing ultrasonic intensity and prolonged treatment time, indicating enhanced lipid degradation. Ultrasound-induced cavitation produces mechanical, thermal, and chemical effects and may alter endogenous enzyme activity [115]. Therefore, amplitude and treatment time should be optimized to balance extraction yield with lipid quality during process scale-up.

### 3.5. Microwave-Assisted Extraction

**Figure 5:**
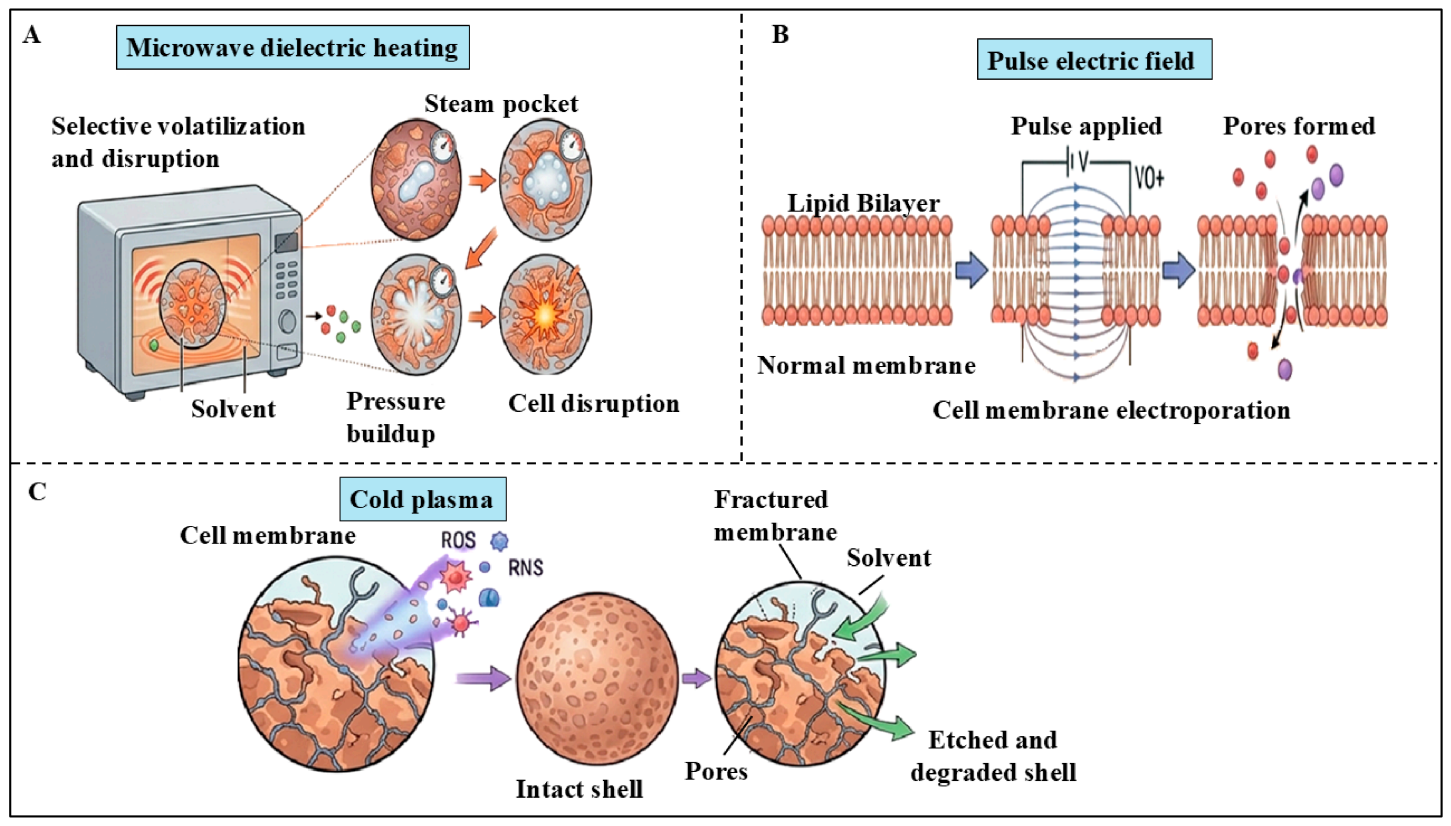
Schematic illustration of extraction mechanisms from crustacean waste using advanced technologies: (**A**) microwave-assisted extraction enhancing cell rupture and mass transfer, (**B**) pulsed electric field (PEF) inducing cell membrane permeabilization, and (**C**) cold plasma generating reactive species for structural disruption.

Another promising method that has been explored in the extraction of various bioactive compounds is microwave-assisted extraction. It is based on dielectric heating, which causes rapid internal heating and pressure buildup within cells, leading to their rupture (Figure 5A). De Oliveira Silva et al. [116] further demonstrated the effectiveness of microwave technology in chitosan production from *Farfantepenaeus brasiliensis* shrimp shells, where microwave-assisted deacetylation achieved a high degree of deacetylation (~90%) and yield (>50%), while significantly reducing reaction time (from 240 to 16 min) and energy consumption (from 382.1 to 8.9 kJ/g). These improvements are attributed to rapid and uniform heating, which enhances reaction efficiency and reduces processing severity. In line with these advancements, Mousavi et al. [117] developed a hybrid process integrating microwave (MW) pretreatment with Soxhlet extraction to enhance the recovery of astaxanthin- and ω-3 PUFA-rich oil from green tiger shrimp (*Penaeus semisulcatus*) residues. Using response surface methodology, optimal MW conditions (400 W, 75 s) were identified, which significantly improved oil yield by promoting structural disruption of shrimp shells, as confirmed by scanning electron microscopy. The study also highlighted the importance of solvent selection, where hexane:isopropanol (1:1, *v*/*v*) achieved the highest oil yield, while hexane:acetone resulted in extracts with superior astaxanthin content, ω-3 PUFA levels, and antioxidant activity. In the chitosan study by De Oliveira Silva et al. [116], life-cycle impact assessment indicated a lower overall environmental impact for microwave-assisted deacetylation than for conventional heating, although NaOH use remained a major contributor. Mohammadi et al. [118] compared conventional and microwave-assisted extraction methods and reported that microwave treatment produced porous, medium-molecular-weight chitosan, whereas conventional extraction yielded high-molecular-weight chitosan and showed stronger overall antibacterial activity. Similarly, El Knidri et al. [119] showed that complete chitosan extraction using microwave irradiation across all steps (demineralization, deproteinization, and deacetylation) reduced processing time from 6 to 7 h to about 24 min while achieving a comparable degree of deacetylation (82%), highlighting significant improvements in efficiency and energy savings. Furthermore, Li et al. [120] introduced a combined microwave and deep eutectic solvent (DES) approach, achieving high chitin yield (19.11%) and purity (97.44%), along with solvent recyclability. Overall, microwave assistance can substantially reduce processing time and energy consumption; however, its industrial sustainability depends on minimizing alkali and organic-solvent use and validating process performance at larger scales [116,117,118,119,120].

### 3.6. Other Advanced Extraction Technologies

Technologies such as pulsed electric field (PEF) and cold plasma treatment operate on distinct but complementary principles that enhance mass transfer and improve extraction efficiency. PEF induces electroporation by applying short high-voltage pulses that create pores in cell membranes, facilitating the release of intracellular compounds (Figure 5B). Gulzar et al. [121] demonstrated that PEF pretreatment of Pacific white shrimp cephalothorax, when followed by UAE, significantly enhanced lipid extraction yield (up to 30.34 g/100 g solids) and reduced lipid oxidation, while improving the recovery of valuable compounds such as PUFAs and carotenoids. Similarly, He et al. [122] reported that high-intensity PEF can be effectively used for chitosan extraction from shrimp shells, achieving a high degree of deacetylation (up to 92.32%) under optimized conditions (field strength 20.48 kV/cm, pulse number 10, and NaOH concentration 48.64%). However, the requirement for 48.64% NaOH indicates that PEF accelerated deacetylation rather than eliminating the use of concentrated alkali. PEF-induced structural disruption may also enhance solvent penetration and mass transfer; however, direct evidence supporting its combination with vacuum impregnation for extracting compounds from crustacean waste remains limited.

In addition, cold plasma pretreatment offers a complementary strategy by inducing surface etching and partial membrane-like structural degradation through the action of reactive oxygen and nitrogen species (RONS) generated in the plasma phase. These reactive species cause oxidation, bond cleavage, and micro-scale erosion of the surface, thereby increasing porosity and exposing reactive sites (Figure 5C). Cai et al. [24] demonstrated that plasma-assisted extraction combined with mild acid–base treatment enables the production of high-purity chitin from Chinese mitten crab shells (*Eriocheir sinensis*) without the need for chemical decolorization, achieving a crystallinity of 64.58%, high thermal stability (DTGmax ≈ 402.9 °C), and a molecular weight of 6.89 × 10^5^ g/mol, comparable to commercial chitin. Building on this, Cai et al. [123] compared conventional alkaline deacetylation with plasma-assisted alkaline deacetylation and reported that plasma-treated samples exhibited higher degrees of deacetylation (65–74%) than the conventional chemical method (58–67%), along with increased crystallinity (up to 31.18%). Additionally, atmospheric-pressure dielectric barrier discharge (DBD) cold plasma has been applied as a pretreatment for crustacean shell waste, achieving intensified protein removal with a reduction of up to 42% [25]. Similarly, nitrogen-based DBD plasma combined with organic acid demineralization (e.g., lactic acid) enabled ~90% protein removal through plasma-generated reactive species that oxidize and denature proteins, disrupt the chitin–protein matrix, and enable complete mineral removal from shrimp shells, highlighting a near-zero-waste approach [26]. Despite these promising results, the application of cold plasma in crustacean waste bioactive extraction remains relatively underexplored. Moreover, oxygen-containing plasma can also degrade the chitin chain under excessive treatment conditions. Therefore, the feed-gas composition and exposure time require careful optimization.

Comparative evaluation of extraction technologies reveals clear differences in efficiency, sustainability, and industrial readiness. Conventional solvent- and acid–alkali-based extraction methods remain widely used due to their simplicity and high recovery yields. However, they suffer from high chemical consumption, environmental burden, and potential degradation of sensitive bioactives. In contrast, ultrasound-assisted extraction and supercritical fluid extraction represent more balanced approaches, offering high extraction efficiency, improved mass transfer, and better scalability, making them among the most promising candidates for near-term industrial application. Emerging green technologies such as deep eutectic solvents, pulsed electric field, and cold plasma provide significant environmental advantages, including reduced solvent toxicity, lower waste generation, and mild operating conditions. However, their industrial translation is still limited by challenges such as high viscosity (DES), energy-demanding equipment (PEF), or incomplete process standardization (plasma-based systems). Microwave-assisted extraction, on the other hand, offers strong process intensification and drastic reduction in extraction time, but requires careful optimization to avoid thermal degradation and ensure product selectivity. Overall, no single extraction technology is universally superior; rather, their performance depends strongly on target compounds, feedstock characteristics, and process conditions. Therefore, future process selection should be guided not only by extraction yield but also by greenness indicators, scalability, energy demand, techno-economic feasibility, and life cycle assessment (LCA)-based evaluation of environmental impacts. Integration of multiple complementary technologies may provide a more sustainable pathway by balancing extraction efficiency, product quality, and industrial feasibility.

## 4. Applications in Various Sectors

Table 2 summarizes representative applications of compounds derived from crustacean by-products in seafood and related industries. The primary compounds obtained include proteins and protein hydrolysates, lipids rich in PUFAs, carotenoids, particularly astaxanthin, and structural biopolymers such as chitin, chitosan, and COS.

### 4.1. Pharmaceutical and Medical Sectors

**Figure 6:**
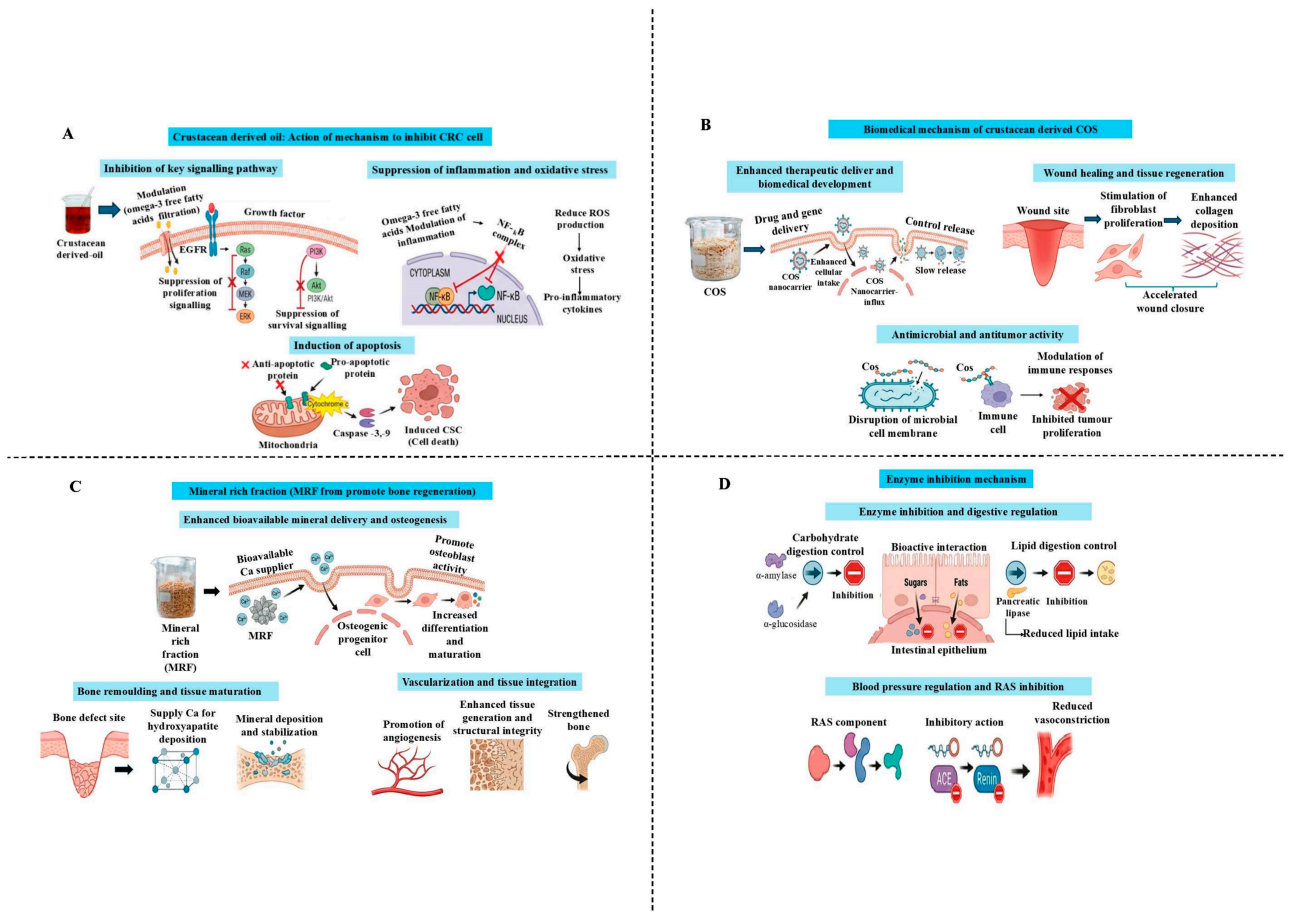
A general description of biomedical applications and mechanisms of crustacean-derived bioactive compounds. (**A**) Anticancer mechanisms of crustacean-derived oils in CRC inhibition. (**B**) Biomedical roles of chitooligosaccharide (COS) including drug delivery, wound healing, and antimicrobial activity. (**C**) Bone regeneration potential of mineral-rich fractions (MRF). (**D**) Enzyme inhibition and metabolic regulation, including carbohydrate digestion and blood pressure control.

Crustacean-derived lipids, particularly krill oil, have demonstrated significant therapeutic potential due to their unique biochemical composition and mechanisms of action. Free fatty acid (FFA) extracts from krill oil have shown strong in vitro antiproliferative activity against colorectal cancer (CRC), reducing the viability of multiple human CRC cell lines by 94–96% at the tested concentrations [124]. This effect was associated with mitochondrial-mediated apoptosis induced mainly by the omega-3 fatty acids EPA and DHA (Figure 6A). In a separate clinical context, Alkhedhairi et al. [125] reported that six-month krill oil supplementation in older adults improved muscle function, including increases in knee extensor strength (9.3%), grip strength (10.9%), and muscle thickness (3.5%), alongside significant elevations in erythrocyte EPA (214%), DHA (36%), and omega-3 index levels (61%). These findings further support the growing use of krill oil as a nutraceutical ingredient in omega-3 dietary supplements aimed at promoting cardiovascular, metabolic, and musculoskeletal health. These benefits are linked to enhanced membrane incorporation of phospholipid-bound omega-3 fatty acids, although the clinical trial did not directly evaluate the underlying mechanisms. Furthermore, a meta-analysis of 14 trials *(n* = 1458) demonstrated that krill oil supplementation significantly reduced total cholesterol, LDL cholesterol, and triglycerides [126], although the meta-analysis did not directly determine the mechanisms responsible for these effects. The presence of astaxanthin further contributes by scavenging reactive oxygen species and protecting cellular components from oxidative damage.

Beyond lipids, crustacean-derived biopolymers such as chitin, chitosan, and COS exhibit diverse biomedical functionalities. COS are widely explored for drug and gene delivery, wound healing, and tissue regeneration due to their biodegradability, mucoadhesive properties, and low toxicity [127]. These properties have facilitated their incorporation into nanoparticle-based drug delivery systems, wound dressings, hydrogels, and tissue-engineering scaffolds. Their mechanisms involve enhanced cellular uptake, controlled release of therapeutic agents, and stimulation of fibroblast proliferation and collagen deposition. Additionally, their antimicrobial and anti-tumor activities are associated with disruption of microbial cell membranes and modulation of immune responses. The antimicrobial activity of COS is mainly due to their positively charged amino groups, which interact with negatively charged microbial cell membranes, leading to increased membrane permeability, leakage of intracellular components, and eventual cell death. They can also bind to microbial DNA, inhibiting mRNA and protein synthesis. Their anti-tumor effects are associated with inhibition of tumor-cell proliferation and metastasis, together with the induction of apoptosis and pro-apoptotic autophagy through the p53/mTOR pathway in an osteosarcoma model (Figure 6B) [128]. Chitin and its derivatives also play important roles in tissue engineering and wound healing, owing to their excellent biocompatibility and ability to form scaffolds that support cell adhesion and regeneration [129]. Mineral-rich fractions from crustacean shells have been investigated as calcium sources for hydroxyapatite production and as calcium supplements in bone-related applications (Figure 6C) [39,130].

**Table 2:** Bioactive compounds from crustaceans and their applications in food industry.

Bioactive Compound	Sector	Application	Key Outcomes	Reference
**Omega-3 and unsaturated fatty acid** **enriched shrimp oil**	Food industry	Emulsion formation. Substitute for soybean oil in salad dressing.	10% incorporation showed the highest consumer acceptance. Improved emulsion stability and viscoelastic properties, product enriched with DHA and EPA.	[28]
**Shrimp shell Chitosan-conjugate**		Bactericidal action against *Listeria monocytogenes*	Exhibited the strongest antibacterial activity among tested conjugates, with MIC and MBC of 1024 µg/mL. At 2 × MIC, it inhibited biofilm formation, reduced extracellular polysaccharide production, and inactivated pre-formed biofilms. MIC/4–MIC inhibited bacterial motility by 85.7–100%.	[131]
**Low cholesterol shrimp lipid-nanoliposomes**		Stabilizing agent in peach tea	Fortification with 3% liposomes resulted in the highest overall acceptability (7.2). Increased polyunsaturated fatty acids and astaxanthin content. Demonstrated improved nutritional value and stability of the beverage.	[132]
**Crab-shell Chitosan**		Antioxidant agent in fish mince	Low-viscosity chitosan (14 cP) reduced hydroperoxide formation by 61% and TBARS by 52% after 8 days of storage. Demonstrated superior inhibition of lipid oxidation compared with higher viscosity chitosan.	[133]
**Chitooligosaccharide–gallic acid conjugate and its liposome**		Act as stabilizing, antioxidant and antibacterial agents in fresh cow milk	Chitooligosaccharide–gallic acid conjugate at 2.50% showed the strongest antimicrobial and antioxidant effects. Reduced lipid oxidation, improved retention of unsaturated fatty acids, and extended milk shelf-life to at least 6 days at 4 °C, compared with less than 4 days for the control.	[134]
**Chitosan-based materials**	Food industry/Packaging	Active packaging for seafood	Films containing 5% tannic acid most effectively reduce lipid oxidation and microbial spoilage. Extended the refrigerated shelf life of baby clam edible portions for up to 12 days through sustained antioxidant and antimicrobial activity.	[135]
**Shrimp shell protein hydrolysates bioactive peptides**	Pharmaceutical and medical	ACE inhibition	Protein hydrolysate produced at 30% DH showed superior bioactivity. The 5–10 kDa peptide fraction exhibited the highest ACE inhibition (44–55%). <1 kDa fraction showed the highest renin inhibition (12–22%), indicating potential for hypertension management.	[57]
**Shrimp shell-derived hydroxyapatite**		Bone regeneration	Calcination at 1000 °C achieved the highest calcium recovery (71.47%). Enabled the production of hydroxyapatite and carbonate hydroxyapatite. Demonstrating the potential of shrimp shell waste as a sustainable source of biomaterials for bone regeneration.	[39]
**Pacific white shrimp (*Litopenaeus vannamei*) shell protein hydrolysates conjugated with polyphenols** **(** **SSPH–PPN)**		α-amylase, α-glucosidase, and pancreatic lipase	Catechin-conjugated hydrolysates showed strong inhibition of α-amylase (89.29%), α-glucosidase (81.23%), and pancreatic lipase (80.69%).	[97]
***Litopenaeus vannamei*** **shell-derived waste chitooligosaccharide conjugated with catechin**		Low-glycemic functional food ingredient/antidiabetic agent	COS-EGCG exhibited strong α-amylase inhibition with IC_50_ of 4.62 mg/mL, lower than COS or EGCG alone. Indicated enhanced inhibitory activity after conjugation.	[98]
**Crab shell-derived calcium and phosphorus along with proteins, amino acids, and chitin.**		Dietary supplement to improve serum calcium and phosphorus levels and Reduced alkaline phosphatase activity in children with rickets	Supplementation significantly increased serum calcium and phosphorus levels Reduced alkaline phosphatase activity. Achieved a 94.3% healing rate, comparable to calcium lactate, reduced concomitant disorders.	[130]
***Litopenaeus vannamei*** **shell-derived waste chitooligosaccharide conjugated with EGCG**		ACE and renin inhibition	Strongest ACE and renin inhibitory activity among tested conjugates. Reduced blood pressure by 21 mmHg in hypertensive rats after 6 h.	[136]
**Pacific blue crab (*Portunus segnis*) protein hydrolysate**		ACE inhibition	Protease production was optimized to 8809 U/mL using 75 g/L crab by-product powder. Protein hydrolysate exhibited ACE inhibitory activity and radical scavenging capacity	[137]
**Crab shell-derived calcium-rich biochar**	Environmental and agricultural	Wastewater treatment (dye adsorption)	Adsorption capacities of 12,502 mg/g for malachite green and 20,317 mg/g for Congo red. Rapid adsorption equilibrium achieved within minutes.	[138]
**Chitooligosaccharides (COS)**		Agricultural biostimulant	Activated jasmonic acid and salicylic acid signaling pathways. Enhanced pathogen resistance and promoted plant growth.	[139]

Crustacean-derived bioactives further exhibit potential in managing metabolic disorders through enzyme inhibition mechanisms. Polyphenol-conjugated shrimp shell protein hydrolysates have shown concentration-dependent in vitro inhibitory activity against α-amylase, α-glucosidase, and pancreatic lipase, mainly by binding to their active or allosteric sites through hydrogen bonding and hydrophobic interactions. These interaction block substrate access and can alter enzyme conformation, reducing catalytic efficiency [97]. Although interactions with enzyme active sites have been proposed, the precise binding modes were not directly established. Functionally, inhibition of α-glucosidase and α-amylase may slow carbohydrate breakdown, whereas pancreatic-lipase inhibition may reduce triglyceride hydrolysis, potentially limiting carbohydrate and lipid digestion. Similarly, EGCG- and gallic-acid-conjugated COS, together with shrimp-shell protein hydrolysates and peptide fractions, demonstrated ACE and renin inhibitory activities [57]. The COS conjugates displayed mixed-type inhibition; however, the precise molecular interactions of the shrimp-shell peptides require further confirmation. ACE inhibition prevents the conversion of angiotensin I to angiotensin II and renin inhibition reduces the formation of angiotensin I. Collectively, these preclinical findings suggest potential blood-pressure-regulating effects through modulation of the renin–angiotensin system, although clinical validation remains necessary (Figure 6D) [57].

### 4.2. Food Industry

The extracted bioactive compounds have been used as antioxidant and antibacterial agents in various foods (fish, bivalves, shrimps, etc.) as well as in different food formulations such as emulsions and foam-based products [140]. Many of these compounds are currently being investigated as natural alternatives to synthetic preservatives and food additives, aligning with the increasing consumer demand for clean-label food products. Chitosan from Tunisian crustacean species was reported to have strong antimicrobial activity, with minimum inhibitory concentrations ranging from 0.156 to 5 mg/mL, against bacterial strains including *Escherichia coli*, *Pseudomonas aeruginosa*, and *Staphylococcus aureus*, as well as antifungal activity against *Candida* species. The chitosan inhibited most of the tested fungi, except *Candida krusei*. Moreover, antioxidant activity was confirmed through DPPH radical scavenging and inhibition of linoleic acid peroxidation, with chitosan from *Parapenaeus longirostris* showing the highest radical scavenging capacity [141]. Because chitosan is insoluble in water, COS has been produced and has shown higher antioxidant and antibacterial activities. However, further enhancement of bioactivity has been achieved through functional modification of chitosan and COS. Conjugation of chitosan and COS with polyphenols, including catechin, epigallocatechin gallate (EGCG), gallic acid, caffeic acid, and ferulic acid, significantly improved antimicrobial properties. Among these, COS–EGCG exhibited the strongest antibacterial activity against *Listeria monocytogenes*, with both minimum inhibitory concentration (MIC) and minimum bactericidal concentration (MBC) of 1024 µg/mL. While low concentrations promoted bacterial growth and biofilm formation, higher concentrations (2 × MIC) effectively inhibited biofilm formation, reduced extracellular polysaccharide production, and inactivated pre-formed biofilms after 48 h. Additionally, COS–EGCG significantly inhibited bacterial motility (85.7–94.3% at MIC/4 and complete inhibition at MIC) [131]. Chitosan and COS exhibit antibacterial activity mainly due to their polycationic nature, which enables electrostatic interaction with negatively charged microbial cell membranes, leading to increased permeability and leakage of intracellular components. They can also chelate essential metal ions, thereby inhibiting microbial enzyme systems and growth. Low molecular weight COS may penetrate the cell and interfere with DNA transcription and protein synthesis. In addition, chitosan forms a protective film on microbial surfaces, restricting nutrient transport. Regarding antioxidant activity, chitosan and its derivatives act as free radical scavengers by donating hydrogen atoms or electrons through their amino and hydroxyl groups. They also chelate transition metal ions, preventing radical generation, and inhibit lipid peroxidation by interrupting oxidative chain reactions [142]. In addition to chitosan and COS, shrimp-derived oils are particularly valuable in formulation development. Gautam et al. [28] reported that omega-3 and unsaturated fatty acid–rich shrimp oil can be incorporated into emulsion-based systems such as salad dressings as a substitute for conventional vegetable oils. The study evaluated incorporation levels of 5%, 10%, 15%, and 20%, revealing that 10% achieved the highest consumer acceptance by balancing nutritional enrichment and sensory quality. Lower levels (5%) provided limited functional differentiation, whereas higher levels (15–20%) reduced acceptability due to the development of fishy flavor and odor associated with marine lipids. Similarly, Raju et al. [132] reported the use of pectin/glycerol-stabilized liposomes loaded with cholesterol-lowered shrimp lipid as fortifying ingredients in beverages such as peach tea. Incorporation at 1%, 3%, and 5% showed that 3% achieved the highest overall acceptability, whereas the lower acceptability at 5% indicated sensory limitations at excessive fortification levels. These findings collectively emphasize that optimal concentration is critical to balance functional benefits and sensory quality in food applications.

Beyond direct incorporation, chitosan and its derivatives have been extensively explored in food preservation and packaging systems, including edible coatings, antimicrobial films, and active packaging materials, due to their antimicrobial and film-forming properties. For instance, Kamil et al. [133] demonstrated that crab shell chitosan effectively reduced oxidative deterioration in fish mince. Among different viscosities tested (14, 57, and 360 cP), low-viscosity chitosan (14 cP) showed superior antioxidant activity, reducing hydroperoxide formation and TBARS by 61% and 52%, respectively, after 8 days of storage. This highlights the importance of molecular characteristics in determining antioxidant efficiency. Advanced chitosan derivatives have further expanded applications in food systems. A COS–gallic acid conjugate (CGC) and its liposomal form (CGLP) have been evaluated as stabilizers in fortified milk systems [134]. At concentrations of 1.25% and 2.50% (*w*/*v*), CGC-2.50 showed the most pronounced antimicrobial and antioxidant effects, significantly reducing microbial counts and lipid oxidation while enhancing retention of unsaturated fatty acids. However, this concentration also altered physicochemical properties, including increased acidity and reduced pH and lightness, indicating the need for optimization to balance functionality and sensory attributes. The effectiveness of crustacean-derived compounds in food systems is largely governed by their antioxidant, antimicrobial, emulsifying, and film-forming mechanisms. These functional properties enable their application as natural preservatives, stabilizers, encapsulation materials, and packaging components that improve food quality, safety, and shelf life while reducing dependence on synthetic additives.

Recent advancements in active packaging systems have further enhanced the application potential of chitosan-based materials. Ponnusamy et al. [135] developed chitosan bilayer films incorporating tannic acid (1–5%), which improved mechanical strength, UV-blocking ability, and antioxidant activity. Films with 5% tannic acid were most effective in reducing microbial spoilage and lipid oxidation in seafood during refrigerated storage, demonstrating sustained antimicrobial action through controlled release mechanisms. Further improvements in seafood preservation have been achieved using chitosan–polyphenol composite films. 

### 4.3. Other Applications

Crustacean-derived compounds have also demonstrated environmental applications. Calcium-rich biochar derived from crab shells has shown high phosphate adsorption capacity, making it suitable for wastewater treatment and environmental remediation. Dai et al. [138] reported that calcium-rich biochar derived from crab shells via a simple pyrolysis process exhibited exceptional adsorption performance for dye removal, with remarkably high adsorption capacities of 12,502 mg/g for malachite green and 20,317 mg/g for Congo red, along with rapid adsorption equilibrium achieved within minutes. The high efficiency was attributed to multiple mechanisms, including electrostatic interactions, hydrogen bonding, and π–π interactions, highlighting its potential as a low-cost and highly effective adsorbent for wastewater treatment. However, its regeneration, performance in complex industrial wastewater, and process economics require further validation. In agriculture, COS functions as bio stimulants by activating jasmonic acid and salicylic acid signaling pathways, enhancing resistance to pathogens and promoting plant growth [139]. From a circular economy perspective, the integration of these valorization pathways enables the transformation of crustacean waste into a wide range of value-added products, thereby reducing environmental burden while enhancing economic returns. The environmental applications of crustacean-derived materials are largely attributed to their high calcium content, porous structure, and abundance of reactive functional groups that facilitate adsorption, ion exchange, and nutrient release. These characteristics make them promising candidates for wastewater treatment adsorbents, biostimulants, nutrient recovery systems, and other sustainable environmental technologies, supporting the transition toward a circular bioeconomy.

Although numerous studies have demonstrated the bioactivity and functional potential of crustacean-derived compounds, their large-scale commercialization remains dependent on several factors, including extraction cost, process scalability, regulatory approval, product stability, and consumer acceptance. For food and packaging applications, feedstock standardization, residual allergen and contaminant control, toxicological assessment, and packaging-migration testing must also be addressed. Nevertheless, krill oil supplements and selected chitosan-based medical and dietary products are commercially available, whereas most crustacean-derived edible coatings and active packaging systems remain at the research or pilot stage, or have achieved limited commercialization. Future developments integrating sustainable extraction and formulation strategies should be supported by techno-economic analysis, life-cycle assessment, safety evaluation, and region-specific regulatory validation.

## 5. Trends and Future Perspectives

Recent trends in crustacean biowaste valorization are increasingly directed toward the development of green, efficient, and integrated extraction technologies for the recovery of high-value biomolecules, including proteins and peptides, chitin and chitosan, lipids, carotenoids, minerals, and industrial enzymes. Emerging approaches such as ultrasound-assisted extraction, microwave-assisted extraction, high-pressure extraction, pulsed electric field processing, cold plasma treatment, supercritical fluid extraction, and deep eutectic solvent-based systems have shown the potential to improve extraction efficiency and selectivity while reducing processing time and chemical consumption under optimized laboratory conditions. Nevertheless, the performance of these technologies varies considerably depending on the target compound, raw material characteristics, operating conditions, and downstream processing requirements. A comparison of the reported studies indicates that conventional chemical extraction remains the most widely applied approach due to its simplicity, high recovery yields, and technological maturity. However, the extensive use of strong acids, alkalis, and organic solvents raises concerns regarding environmental impact, product quality degradation, and waste generation. In contrast, emerging green technologies generally offer improved sustainability and reduced chemical usage but often require specialized equipment, higher capital investment, and further validation at pilot and industrial scales. However, the designation of an extraction technology as “green” should be supported by quantitative sustainability assessments rather than qualitative descriptions alone. Future studies should incorporate standardized evaluation tools, including life cycle assessment (LCA), techno-economic analysis (TEA), energy consumption analysis, carbon footprint estimation, and solvent toxicity assessment, to objectively determine the environmental and economic performance of extraction processes. The application of such metrics would facilitate direct comparisons among conventional and emerging extraction technologies and support informed decision-making for industrial implementation. Similarly, enzyme-assisted processes provide high selectivity and product quality under mild conditions but are frequently constrained by enzyme cost, process duration, and operational complexity. These observations highlight that no single extraction strategy is universally optimal, and technology selection should be based on the desired product, purity requirements, economic feasibility, and intended application. Despite substantial advances, several research gaps remain. Many studies focus primarily on extraction yield and physicochemical characterization while providing limited information regarding process scalability, reproducibility, energy consumption, solvent recovery, and long-term economic feasibility. Furthermore, direct comparisons among extraction technologies are often difficult due to variations in raw material sources, extraction conditions, analytical methodologies, and reporting standards. Future investigations should therefore adopt standardized protocols and performance indicators to facilitate meaningful comparisons between studies and accelerate technology transfer. In addition, greater emphasis should be placed on developing standardized reporting frameworks to improve data comparability and reproducibility across laboratories.

Future research should move beyond laboratory-scale extraction efficiency and focus on process intensification, integrated biorefinery concepts, and cascade utilization strategies that enable the simultaneous recovery of multiple bioactive compounds from a single biomass stream. Such approaches can maximize resource utilization, reduce waste generation, and improve the overall economic viability of crustacean biorefineries. From the perspective of industrial implementation, extraction technologies should be evaluated not only based on recovery efficiency but also on technology readiness, economic feasibility, and regulatory suitability. Among the emerging approaches, ultrasound-assisted extraction appears most suitable for near-term adoption as a scalable pretreatment or process-intensification step. Enzyme-assisted extraction is particularly relevant to high-value products for which improved selectivity can offset enzyme costs, whereas supercritical fluid extraction is technologically advanced for lipids and carotenoids but remains constrained by high-pressure equipment costs and, for polar compounds, the need for co-solvents. DES, PEF, and plasma-based processes require more extensive pilot-scale validation before their industrial readiness can be established. Nevertheless, challenges related to equipment investment, energy demand, enzyme cost, process complexity, and reproducibility must be addressed to ensure economic viability. Furthermore, successful commercialization requires compliance with regulatory requirements through standardized quality assessment, contaminant monitoring, safety evaluation, and consistent characterization of recovered compounds. Consequently, near-term industrial adoption is most likely to involve target-specific hybrid processes that combine established operations with selected green pretreatments, rather than the complete replacement of conventional processing by a single emerging technology.

Another important challenge relates to product safety and regulatory compliance. Crustaceans are known to bioaccumulate environmental contaminants, including heavy metals such as lead, cadmium, mercury, and cobalt, depending on species, habitat, and feeding behavior [143]. These contaminants may be co-extracted with target compounds and potentially affect product quality and safety, particularly for food, nutraceutical, pharmaceutical, and biomedical applications. Consequently, future scale-up strategies should incorporate robust purification processes, routine contaminant monitoring, and strict quality-control measures. Validated techniques such as inductively coupled plasma mass spectrometry (ICP-MS) or atomic absorption spectroscopy (AAS) should be selected according to the target element, sample matrix, required detection limit, and applicable regulatory standards. Safety assessment should also address residual shellfish allergens, microorganisms, extraction-chemical residues, and solvent migration where relevant. Overall, the future of crustacean biowaste valorization lies in the integration of green extraction technologies, comprehensive safety assessment, standardized process evaluation, and economically viable biorefinery models. The immediate research priority is therefore the pilot-scale validation of target-specific-hybrid biorefineries supported by complete mass and energy balances, solvent and enzyme recovery data, product-safety testing, TEA, LCA, and market-specific regulatory pathways. Such evidence is essential for bridging the gap between laboratory research and safe, economically viable commercial production within a circular bioeconomy.

## 6. Conclusions

Crustacean waste, once regarded merely as an environmental burden, is increasingly being recognized as a sustainable and renewable source of bioactive and functional compounds. Generated in large quantities from shrimp, crab, and lobster processing industries, this waste represents a rich reservoir of valuable materials such as chitin, proteins, lipids, omega-3 fatty acids, and the potent antioxidant astaxanthin. Advances in extraction and purification technologies, particularly non-thermal and enzyme-assisted methods, have enabled these compounds to be recovered with improved efficiency and selectivity and potentially lower environmental impacts. Among the technologies reviewed, ultrasound-assisted extraction, enzyme-assisted processes, and supercritical fluid extraction emerge as the most promising for near-term industrial implementation due to their relatively high extraction efficiency, improved selectivity, reduced solvent usage, and demonstrated scalability. In contrast, emerging approaches such as deep eutectic solvent-based systems, pulsed electric field processing, and cold plasma treatment show significant potential but still require further optimization, techno-economic validation, and pilot-scale demonstration before full commercialization. These approaches can reduce chemical consumption and process severity while helping to preserve the structural integrity and biological activity of recovered compounds. However, reductions in energy consumption and overall environmental impacts must be confirmed through quantitative assessment before industrial implementation. The valorization of crustacean by-products contributes significantly to waste reduction, pollution mitigation, and resource recovery, thereby supporting the principles of a circular bioeconomy and sustainable blue growth. While conventional chemical-assisted processes remain relevant due to their simplicity and effectiveness, industrial transition is likely to rely on hybrid biorefineries that combine established operations with target-specific greener processes. Such biorefineries should enable the sequential recovery of multiple products from a single feedstock, thereby improving resource efficiency and economic viability. Furthermore, successful industrial translation will require standardized process evaluation, techno-economic feasibility studies, life cycle assessment, and strict quality and safety controls, particularly considering the possible co-extraction of environmental contaminants such as potentially toxic elements. In addition, the development of standardized product specifications and harmonized regulatory frameworks will be essential to ensure product quality, safety, and consumer acceptance across different application sectors. In summary, the future success of crustacean waste valorization will depend not only on improving extraction efficiency but also on achieving a balance between technological performance, environmental sustainability, economic feasibility, and industrial practicality. The most suitable approach should therefore be selected based on a holistic evaluation of product requirements, processing constraints, and sustainability targets. Moving from laboratory-scale investigations to commercially viable applications will require pilot-scale validation, reproducible product specifications, and stronger collaboration among researchers, regulators, and industry. The central priority is therefore to convert crustacean processing residues into safe, standardized, and market-relevant products while demonstrating environmental and economic benefits at pilot and industrial scales. Achieving this objective would strengthen resource efficiency and support the commercialization of sustainable bioproducts, and sustainable innovation across the seafood and bioproduct sectors.

## Data Availability

All data supporting the findings of this study are openly accessible within the published article.
